# Estimating spatially variable and density‐dependent survival using open‐population spatial capture–recapture models

**DOI:** 10.1002/ecy.3934

**Published:** 2023-01-05

**Authors:** Cyril Milleret, Soumen Dey, Pierre Dupont, Henrik Brøseth, Daniel Turek, Perry de Valpine, Richard Bischof

**Affiliations:** ^1^ Faculty of Environmental Sciences and Natural Resource Management Norwegian University of Life Sciences Ås Norway; ^2^ Norwegian Institute for Nature Research (NINA) Trondheim Norway; ^3^ Department of Mathematics and Statistics Williams College Williamstown Massachusetts USA; ^4^ Department of Environmental Science, Policy and Management University of California Berkeley Berkeley California USA

**Keywords:** mortality, nimbleSCR, population dynamics, population‐level inferences, wolverines (*Gulo gulo*)

## Abstract

Open‐population spatial capture–recapture (OPSCR) models use the spatial information contained in individual detections collected over multiple consecutive occasions to estimate not only occasion‐specific density, but also demographic parameters. OPSCR models can also estimate spatial variation in vital rates, but such models are neither widely used nor thoroughly tested. We developed a Bayesian OPSCR model that not only accounts for spatial variation in survival using spatial covariates but also estimates local density‐dependent effects on survival within a unified framework. Using simulations, we show that OPSCR models provide sound inferences on the effect of spatial covariates on survival, including multiple competing sources of mortality, each with potentially different spatial determinants. Estimation of local density‐dependent survival was possible but required more data due to the greater complexity of the model. Not accounting for spatial heterogeneity in survival led to up to 10% positive bias in abundance estimates. We provide an empirical demonstration of the model by estimating the effect of country and density on cause‐specific mortality of female wolverines (*Gulo gulo*) in central Sweden and Norway. The ability to make population‐level inferences on spatial variation in survival is an essential step toward a fully spatially explicit OPSCR model capable of disentangling the role of multiple spatial drivers of population dynamics.

## INTRODUCTION

Spatial capture–recapture (SCR) models are hierarchical models that use the spatial information contained in repeated individual detections to account for imperfect detection and estimate density (Borchers & Efford, 2008; Efford, 2004; Royle et al., 2014). Because SCR models are spatially explicit and accommodate various types of data (e.g., physical capture, photographic, noninvasive genetic, or acoustic detection), they are now routinely used to analyze wildlife monitoring data. When data are collected over several consecutive occasions, open‐population SCR (OPSCR) models can be used to estimate demographic rates and movement of individuals between occasions in addition to densities (Bischof, Milleret, et al., 2020). Modeling individual movement between occasions helps return unbiased estimates of survival (Gardner et al., 2018), but see Efford and Schofield (2022). These properties make OPSCR models well suited for drawing population‐level inferences about the drivers of demographic processes.

Demographic rates, such as survival, are known to vary over time (Gaillard et al., 2000), with individual attributes (de Valpine et al., 2014), and across space (DeCesare et al., 2014). Temporal and individual variation of demographic parameters can be readily integrated into OPSCR models (Bischof, Milleret, et al., 2020), and the possibility of inferring spatial heterogeneity in survival using OPSCR models has been suggested (Royle et al., 2014) and applied (Chandler et al., 2018). However, the performance of models that estimate spatially variable survival has not been thoroughly tested, and such models’ potential remains underexploited. Estimation of spatially varying vital rates is a key step in the development of OPSCR models because it will lead to a better understanding of the processes driving the spatial distribution of individuals (Royle et al., 2014).

At their core, OPSCR models account for imperfect detection using an observation process that assumes that an individual's detection probability is a function of distance from its activity center (AC) (Borchers & Efford, 2008; Royle et al., 2014). The location of individual ACs is a latent variable and represents the center of an individual's home range. AC locations are a key quantity of OPSCR models because they allow for the estimation of density and interannual movement. ACs also provide the spatial information necessary to characterize the environment surrounding individuals and, therefore, its influence on survival (Chandler et al., 2018).

Density is a key driver of survival (Gaillard et al., 2000), but studies on density‐dependent survival have often been limited to estimating the average population response to variation in overall population size through time (Bonenfant et al., 2009). However, variation in density is a spatiotemporal process, and individuals within a population may not experience the same density. By estimating spatiotemporal variation in density, OPSCR models offer a unique opportunity to study density dependence in survival at the local scale, while accounting for variation and uncertainty in both density and survival within a unified framework.

Here, we present a Bayesian OPSCR model that accounts for spatial variation in survival as a function of spatial covariates (e.g., characteristics of the landscape, resource availability) and density. We model survival using a hazard rate formulation to allow for inferences on spatial variation in competing risks of mortality (Ergon et al., 2018). We quantify model performance by simulating OPSCR data sets for a wide range of scenarios. In addition, we quantify the consequence of ignoring spatial heterogeneity in mortality for OPSCR inferences. As an empirical demonstration, we use the model to estimate the effect of density and country on cause‐specific mortality of female wolverines (*Gulo gulo*) in central Sweden and Norway. All functionalities are made available in the R package nimbleSCR, which provides tools for fitting efficient Bayesian (Markov chain Monte Carlo [MCMC]) models (Bischof, Turek, et al., 2020; de Valpine et al., 2017; NIMBLE Development Team, 2019; Turek et al., 2021).

## METHODS

### OPSCR model

To estimate spatial variation in mortality from live encounters and dead recoveries collected over several consecutive occasions (hereafter “years”), we built a Bayesian hierarchical state‐space OPSCR model. The model is composed of four submodels for (1) density and interannual movement, (2) demography, (3) live detections, and (4) dead recoveries (Bischof, Milleret, et al., 2020; Dupont et al., 2021; Milleret et al., 2020, 2021; Sun et al., 2014). We created two versions of the model. The first version can distinguish between two spatially variable and competing causes of mortality. For example, it is possible to distinguish between legal culling and other causes of mortality in cases where all individuals culled are recovered dead (Bischof, Milleret, et al., 2020). The second model only considers one cause of mortality. This reflects a realistic limitation because dead recoveries are not always available in OPSCR data sets, and distinguishing between multiple causes of death may not be possible.

#### Spatial distribution and movement submodel

In SCR models, the location of individuals is represented by their ACs within the spatial domain (*S*). We used a Bernoulli point process with spatial intensity $\lambda \left(s\right)$ to model the distribution of ACs, where ***s***_***i,t***_ is the vector of *x* and *y* spatial coordinates of individual *i*'s AC at time *t* (Zhang et al., 2022). For *t* > 1, the probability density of $s_{i,t}$ is conditional on the Euclidean distance to $s_{i,t-1}$:

(1) $$\left(s_{i,t}|s_{i,t-1},\tau \right)\propto e^{-\frac{{\left\|s_{i,t}-s_{i,t-1}\right\|}^{2}}{2\tau^{2}}},$$

where τ is the standard deviation of a bivariate normal distribution centered on $s_{i,t-1}$ and represents movement between *t* – 1 and *t* (Gardner et al., 2018).

#### Demographic submodel

We used a multistate formulation (Lebreton & Pradel, 2002) where each individual life history is represented by a succession of up to four discrete states *z*_*i*,*t*_: (1) “unborn” if the individual has not been recruited in the population, (2) “alive” if it is alive, (3) “culled” if it was culled and recovered dead between the start of the previous and current occasion, or (4) “dead” if it has died but was not recovered dead or died earlier, regardless of the cause. We used data augmentation, whereby additional, undetected individuals are available for inclusion in the population at each time step (Royle et al., 2007).

During the first year, individuals can only be designated as “unborn” (*z*_*i*,1_ = 1) or “alive” (*z*_*i*,1_ = 2) so that *z*_*i*,1_ ~ dcat(1 − $\gamma_{1}$, $\gamma_{1},0,0$), where $\gamma_{1}$ represents the probability of being “alive” at time *t* = 1.

For *t* ≥ 2, *z*_*i*,*t*_ is conditional on the state of individual *i* at *t* − 1:

1. If *z*_*i*,*t*–1_ = 1, individual *i* can be recruited (i.e., transition to state 2) with probability $\;\gamma_{t}$ or remain unborn with probability 1 − $\gamma_{t}$, so *z*_*i*,*t*_ ~ dcat(1 $-\gamma_{t},\gamma_{t},0,0)$.
2. If *z*_*i*,*t*–1_ = 2, individual *i* can survive with probability $\phi_{i}$ and remain *z*_*i*,*t*_ = 2. If it does not survive, it can either die due to culling and be recovered (transition to *z*_*i*,*t*_ = 3) with probability *h*_*i*_ or die from other causes without being recovered (transition to *z*_*i*,*t*_ = 4) with probability *w*_*i*_, so that *z*_*i*,*t*_ ~ dcat(0, $\phi_{i}$, *h*_*i*_, *w*_*i*_), where Φ_*i*_ = 1 − *h*_*i*_ − *w*_*i*_.
3. All individuals in dead states (*z*_*i*,*t*–1_ = 3 or 4) transition to *z*_*i*,*t*–1_ = 4, the absorbing state, with probability 1, so that *z*_*i*,*t*_ ~ dcat(0, 0, 0, 1)

Abundance estimates are obtained by ${\hat{N}}_{t}=\sum_{i=1}^{M}I\left(z_{i,t}=2\right)$, where $I\left(z_{i,t}=2\;\right)$ is an indicator function to count alive individuals, and *M* is the number of detected and augmented individuals.

##### Mortality hazard rates

When an individual dies from a specific cause, it is no longer available to die from another cause. Mortality causes are competing and nonindependent; we therefore parameterized the model using mortality hazard rates (Ergon et al., 2018). We expressed individual survival and mortality probabilities as functions of the culling hazard rate ($m_{h_{i}}$) and the hazard rate associated with all other causes of mortality ($m_{w_{i}}$). For simplicity, we assumed that the hazard rate from culling and other causes remained proportional within time intervals

(2) $$\phi_{i}=\exp\left(-\left(m_{h_{i}}+{m_{w}}_{i}\right)\right),$$

(3) $$h_{i}=\left(1-\phi_{i}\right)\;\left(\frac{m_{h_{i}}}{m_{w_{i}}+{m_{w}}_{i}}\right),$$

(4) $$w_{i}=\left(1-\phi_{i}\right)\;\left(\frac{\;{m_{w}}_{i}}{m_{h_{i}}+{m_{w}}_{i}}\right).$$

##### Spatial variation in mortality

We accounted for spatial variation in cause‐specific mortality by modeling individual mortality hazard rates ($m_{h_{i}}$ and $m_{w_{i}}$) as functions of a spatial covariate ${\text{SpatialCov}}_{s_{i,t}}$ at the location of the individual AC $\left(s_{i,t}\right)$:

(5) $$\log\left(m_{h_{i,t}}\right)=\log\left(m_{0h}\right)+\beta_{h}\times {\text{SpatialCov}}_{s_{i,t}},$$

(6) $$\log\left(m_{w_{i,t}}\right)=\log\left(m_{0w}\right)+\beta_{w}\times {\text{SpatialCov}}_{s_{i,t}}.$$

Here $\beta_{h}$ and $\beta_{w}$ are the coefficients of the relationship between the spatial covariate on culling and other mortality, respectively. Parameters $m_{0h}$ and $m_{0w}$ represent the baseline culling and other mortality hazard rates, respectively.

##### Density dependent survival

At each occasion *t*, local density within any habitat cell *r* of *S* (*r* = 1, …, *R*) can be obtained as $\;d_{r,t}$ = $\sum_{i=1}^{M}I\left(z_{i,t}=2,s_{i,t}=r\right)$, where $I\left(z_{i,t}=2,s_{i,t}=r\right)$ is an indicator function denoting whether the individual is alive and its AC falls within cell *r*.

By replacing the SpatialCov in Equations (5) and (6) with the logarithm of the local density, log($d_{s_{i,t},t})$, we can estimate the effect of local density dependence on individual mortality between occasion (*t* − 1) and *t*. Note that since Equations (5) and (6) are on the log scale, transformation of $d_{r,t}$, such as $d_{r,t}^{\prime }=f\left(d_{r,t}\right)=d_{r,t}+1$, is necessary to avoid $\hat{d_{r,t}}=0$.

#### Live detection submodel

We used the half‐normal function to model the detection probability of individuals alive, whereby the probability *p*_*i,j,t*_ of detecting individual *i* at detector *j* and time *t* decreases with distance between the location x of detector *j* and AC (***s***_*i,t*_):

(7) $$p_{i,j,t}=p_{0}\times \exp\left(-\frac{1}{2\sigma^{2}}{\left\|s_{i,t}-x_{j}\right\|}^{2}\right),$$

where *p*_0_ is the baseline detection probability, and σ is the scale parameter. The detection *y*_*i,j,t*_ of alive individual *i* at detector *j* on time *t* is modeled as the realization of a Bernoulli process conditional on both the “alive” individual state (i.e., *z*_*i*,*t*_ = 2) and the individual and detector‐specific detection probability *p*_*i,j,t*_:

(8) $$y_{i,j,t}∼\text{Bernoulli}\left(p_{i,j,t}\times I\left(z_{i,t}=2\;\right)\right).$$

#### Dead recovery model

We used a Bernoulli point process with a bivariate normal model to model dead recoveries within *S*:

(9) $$\lambda \left(y.{\text{dead}}_{i,t}|s_{i,t},z_{i,t},\sigma \right)\propto e^{-\frac{{\left\|y.{\text{dead}}_{i,t}-s_{i,t}\right\|}^{2}}{2\sigma^{2}}}\times I\left(z_{i,t}=3\right),$$

where *y.dead*_*i,t*_ is the vector of spatial coordinates of the dead recovery location for individual *i* at time *t*. The indicator function conditions the occurrence of dead recoveries when the individual was culled and recovered dead. The detection probability function represents space use; we therefore assume that σ, the scale parameter of the detection probability function, is identical for live detections and dead recoveries.

### OPSCR model with a single cause of mortality

When dead recoveries (*y.dead*) are not available and cause‐specific mortality is not estimable, we can model three demographic states *z* instead of four. Individuals could be unborn ($z_{i,t}=1$), alive ($z_{i,t}=2$), or dead ($z_{i,t}=3$). For *t* > 1, alive individuals ($z_{i,t-1}=2$) can survive with probability $\phi_{i,t-1}$ and remain $z_{i,t}=2$ or die with probability ($1-\phi_{i,t-1}$) and transition to $z_{i,t}=3$, the dead absorbing state. We modeled the effect of density at occasion *t* on individual survival between occasion *t* and *t* + 1:

(10) $$\log\left(m_{i,t}\right)=\log\left(m_{0}\;\right)+\beta_{d}\times \log\left(d_{t_{s_{i,t}}}+1\right),$$

(11) $$\phi_{i,t}=1-\exp\left(-m_{i,t}\right).$$

### Simulations

We conducted simulations to quantify the performance of (1) the OPSCR model in estimating spatial variation in cause‐specific mortalities with a deterministic spatial covariate and (2) the OPSCR model with integrated density‐dependent survival. Finally, we studied the consequences of ignoring spatial variation in survival for abundance estimates.

#### Using deterministic covariate

**FIGURE 1:**
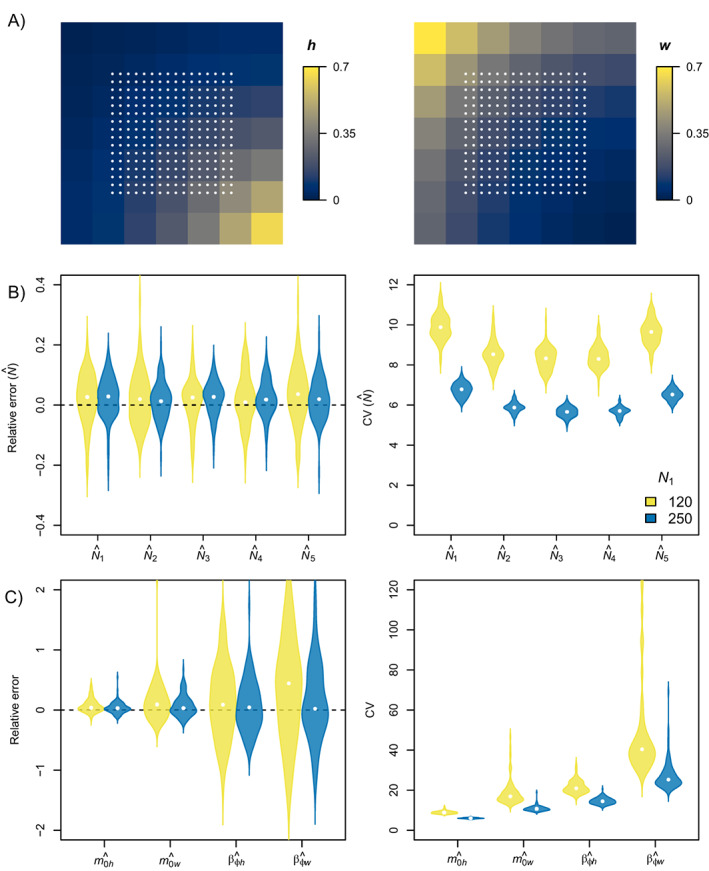
(A) Maps depicting simulated spatial variation in cause‐specific mortality (*h* and *w*) within the spatial domain (S) for the scenario with a gradient covariate $\beta_{h}$ = −1 and $\beta_{w}$ = 1 (Appendix S1: Table S5). White points represent detectors. Violin plots show the distribution of the relative error (left; points: relative bias) and coefficient of variation (CV; right; points: median) in (B) occasion‐specific abundances, and (C) cause‐specific mortality parameters obtained after fitting the open‐population spatial capture–recapture model accounting for spatial variation in mortality to 100 replicated data sets for small (*N*_1_ = 120) and large (*N*_1_ = 250) population size.

We created a spatial domain (*S*) of 28 × 28 distance units (du) subdivided into *R* = 49 cells of 4 × 4 du. We centered in *S* a 16 × 16 detector grid (with a minimum distance of 1 du between detectors). This left a 6 du buffer around the detector grid where individuals cannot be detected alive. We set $p_{0}$ = 0.1, σ = 2, and τ=3 and considered five consecutive occasions. This setup led to an average of two (95% quantiles = 1.5–2.3) detections per individual detected and on average 43% (95% quantiles = 34%–51%) of individuals alive detected on each occasion (Appendix S1: Table S1). We created a spatially autocorrelated covariate following a diagonal gradient (SpatialCov), ranging from −2 to 2 (Figure 1A). We set *M* = 650 and *N*_1_ = 250 individuals on the first occasion, leading to $\gamma_{1}=\frac{N_{1}}{M}=\frac{250}{650}$. For *t* > 1, we assumed a constant 0.3 per capita recruitment rate. We set the baseline mortality hazard rates to $m_{0_{h}}$ = −2.25 and $m_{0_{w}}$= −1.75 and created four different scenarios with all combinations of $\beta_{h}=$ c(1, −1) and $\beta_{w}=$ {1, −1}.

We repeated the simulation scenarios described previously, but with (1) lower population size, *N*_1_ = 120 (*M* = 370), and (2) a spatially random covariate SpatialCov_*r*_ ~ Uniform(−1, 1) (Appendix S1: Table S1). We expected estimation performance for such scenarios to be more challenging due to (1) sparser OPSCR data sets and (2) lower level of spatial autocorrelation and overall variation in mortality. In total, we simulated 100 replicated OPSCR data sets from each of the 16 scenarios (Appendix S1: Table S1). We used NIMBLE's simulation feature (de Valpine et al., 2017; NIMBLE Development Team, 2019) to simulate OPSCR data sets directly from the nimble OPSCR model.

#### Using density‐dependent survival

To illustrate how to estimate density‐dependent survival, we used the OPSCR model with a single source of mortality. Preliminary analyses showed that, computationally (convergence, mixing), OPSCR models performed more poorly when survival was modeled as dependent on latent density rather than deterministic spatial covariates. To counter this, we used a larger spatial domain (*S*) to increase the number of habitat cells (*R*) and, thus, provide the model with more variable latent density points to serve as a covariate on the mortality hazard rate. This led to a habitat of 40 × 40 du subdivided into *R* = 64 cells of dimension 5 × 5 du in which we centered a 30 × 30 detector grid. We set *M* = 650 and *N*_1_ = 250. We set $m_{0}$ = 1.6 and $\beta_{d}=$ −1 to simulate negative density dependence on survival and simulated 100 replicated OPSCR data sets (Appendix S1: Table S12).

#### Ignoring spatial heterogeneity in mortality

To evaluate whether ignoring spatial variation in mortality led to biased parameter estimates, especially abundance ($\hat{N}$), we fitted all simulated data sets described earlier with OPSCR models that assumed constant survival across space and time. As in the earlier simulations, we fitted models with two competing risks for the deterministic spatial covariate scenarios and a single cause of mortality for the density‐dependent scenario.

### Model fitting

We fitted the Bayesian OPSCR models using MCMC simulation with NIMBLE in R version 4.1.0 (R Core Team, 2021). We used the R package nimbleSCR (Bischof, Turek, et al., 2020; Turek et al., 2021), which implements the local evaluation approach (Milleret et al., 2019), to increase MCMC efficiency. For each simulation, we ran three chains of 30,000 (60,000 for density‐dependent survival) iterations, including a 2000‐iteration burn‐in. We considered models as converged when the Gelman‐Rubin diagnostic ($\hat{R}$, Gelman & Rubin, 1992) was <1.1 for all parameters and by visually inspecting traceplots from a randomly selected subset of simulations. Following Gimenez et al. (2009), we also computed the prior‐posterior distribution overlap and used overlap ≥35% as an indicator of weak identifiability.

### Evaluation of model performance

We summarized the posterior $\hat{\theta }$ for each parameter and each simulation using the relative error of the mean posterior $\left(\frac{\bar{\theta }-\theta }{\theta }\right)$ and relative precision as the coefficient of variation $\left(\text{CV}=\frac{\mathrm{SD}(\hat{\theta })}{\bar{\left|\theta \right|}}\right)$, where θ is the true (simulated) parameter value, $\bar{\theta }$ the mean of the posterior, and $\mathrm{SD}(\hat{\theta })$ the standard deviation of the posterior. We quantified the accuracy of the estimators across many simulations using the relative bias of the average of the mean posteriors and a coverage accuracy of 95% credible intervals (CI). The latter was determined as the rate of correct inferences, which is the probability that the 95% credible interval of the parameter estimate contains the true value of that parameter. In addition, we used results from a few of the simulated scenarios with a deterministic covariate (as described previously) to quantify the ability of the model to predict the spatial pattern in mortality (see further details in Appendix S1, Figure S12).

### Empirical example

We obtained data from the noninvasive genetic monitoring of wolverines collected during seven consecutive winters (2014–2020) in central Sweden and Norway (Bischof, Milleret, et al., 2020). We used 2443 genotyped samples (429 individuals) and 126 dead recoveries of ≥1 year old female wolverines (Figure 3A, see Appendix S2: Tables S1–S3 for further details on the sampling). We fitted an OPSCR model, as described in *OPSCR model*, where individuals can either die from culling (all culling events are known and reported; *n* = 118) or from other causes that mostly remain unknown (only eight females were reported to be dead due other causes). We modeled spatial variation in culling $\beta_{h}$ and other causes $\beta_{w}$ of mortalities as a function of country (Norway = 0, Sweden = 1) and local density ($\beta_{{\text{Dens}}_{w}},\beta_{{\text{Dens}}_{h}}$):

(12) $$\begin{array}{c} \log\left(m_{h_{i,t}}\right)=\log\left(m_{0h}\right)+\beta_{h}\times {\text{Country}}_{s_{i,t}}+\beta_{{\text{Dens}}_{h}}\\ \;\times \log\left(d_{t_{s_{i,t}}}+1\right), \end{array}$$

(13) $$\begin{array}{c} \log\left(m_{w_{i,t}}\right)=\log\left(m_{0w}\right)+\beta_{w}\times {\text{Country}}_{s_{i,t}}+\beta_{{\text{Dens}}_{w}}\\ \;\times \log\left(d_{t_{s_{i,t}}}+1\right). \end{array}$$

Wolverine detections were aggregated to the closest detector in a grid of detectors. To account for spatiotemporal heterogeneity in detection probability, we used different covariates, such as the recorded search effort, the presence of snow, and distance to roads (see Appendix S2 and [Bischof, Milleret, et al., 2020] for further details). We ran four chains of 32,500 iterations, including a 10,000‐iteration burn‐in, and followed the same procedure to assess model performance as we did in the simulation study. We present the cause‐specific mortality hazard rates back‐transformed to probabilities to ease the interpretation.

## RESULTS

### Spatial heterogeneity using a deterministic spatial covariate

Scenarios with a spatially random covariate and low population size (*N*_1_ = 120) led to the poorest parameter convergence and identifiability (28%–57% of simulations), whereas all models converged with a spatial gradient covariate and large population size (*N*_1_ = 250; Appendix S1: Table S2). All model parameters had low bias (≤6% relative bias) for all scenarios with a large population size and a gradient covariate (Figure 1, Appendix S1: Tables S3–S6). Accurate estimates were more challenging to obtain for scenarios with a low population size and a spatially random covariate (Appendix S1: Tables S3–S6). For example, the relative bias of $\beta_{w}$ reached 44% for the scenario with low population size (*N*_1_ = 120), whereas there was virtually no bias (RB = 2%) with a larger population size (*N*_1_ = 250; Appendix S1: Table S5). Coverage remained relatively high (>90%) for all scenarios (Appendix S1: Tables S3–S6).

### Density‐dependent survival

**FIGURE 2:**
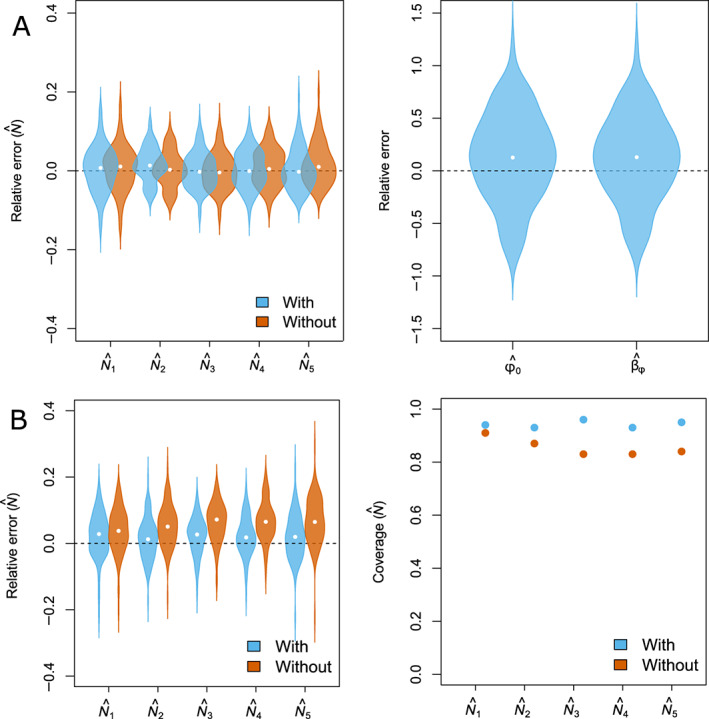
(A) Violin plots (points: medians = relative bias) representing the distribution of the relative error in occasion‐specific abundances ($\hat{N}$) and parameters controlling for the effect of density in survival ($\beta_{\varphi },\varphi_{0}$). Abundance estimates and associated relative error were obtained by fitting an open‐population spatial capture–recapture (OPSCR) model that did not account for spatial variation in survival (“without”) and a model that accounted for density‐dependent survival (“with”) to 100 replicated data sets simulated with a negative effect of density on survival $\beta_{\varphi }$ = −1. (B) Violin plots of distribution of relative error (left; points: medians = relative bias) and coverage (right) of occasion‐specific abundances obtained by fitting an OPSCR model that accounted for spatial variation in mortality (“with”) and a model did not (“without”). Results are presented for 100 replicated data sets simulated with a spatial gradient in mortality, $\beta_{h}$ = −1, $\beta_{w}$ = 1, and *N*_1_ = 250.

Across the 100 replicated data sets, 86 models reached convergence and showed no identifiability issues (Appendix S1: Table S13). We detected a 13% positive relative bias in $\;\phi_{0}$ and $\beta_{d}$, but coverage was >92% for all parameters (Figure 2A, Appendix S1: Table S14).

### Ignoring spatial heterogeneity in mortality

Apart from not being able to provide robust inferences on mortality, not accounting for spatial heterogeneity in mortality can lead to bias in abundance estimates. The relative bias (up to 10% for our simulated scenarios) and lower coverage were especially pronounced for scenarios with a spatial gradient in mortality (Figure 2B, Appendix S1: Tables S7–S11).

### Wolverine empirical example

**FIGURE 3:**
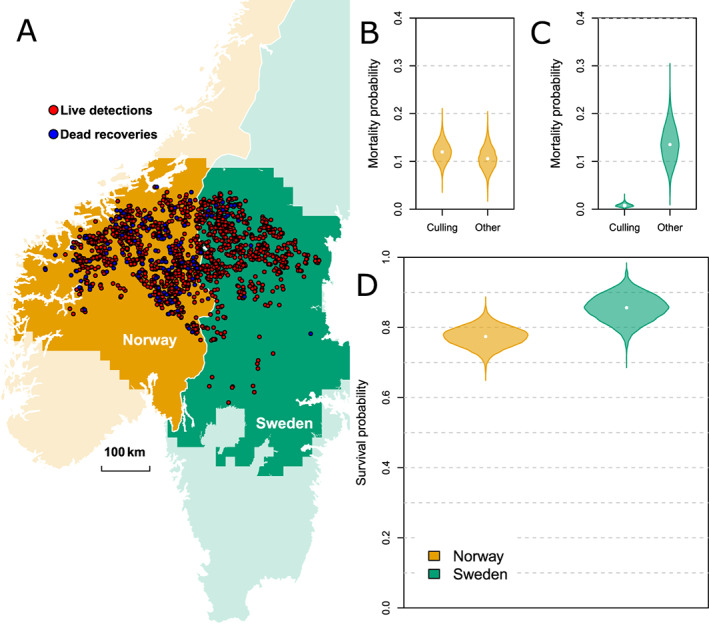
(A) Map showing location of live detections (noninvasive genetic samples; red points) and dead recoveries (blue points) of female wolverines in Norway (yellow) and Sweden (green) from 2014 to 2021. Darker background colors show extent of habitat considered in the analysis. Violins represent posterior distribution of cause‐specific mortalities due to culling and all other causes in (B) Norway and (C) Sweden, and (D) overall survival probabilities in Norway and Sweden.

We found that culling mortality of female wolverines was lower in Sweden than in Norway ($\beta_{h}=-2.76;95\%\mathrm{CI}:\left[-2.08;-3.56\right]$), but mortality due to other causes was not significantly different between the countries ($\beta_{w}=0.18;\left[-0.36;-0.65\right]$; Figure 3, Appendix S2: Figure S4). Mortality due to culling ($\beta_{\mathrm{Den}s_{h}}=0.70;\left[-0.75;2.08\right]$) and to other causes ($\beta_{\mathrm{Den}s_{h}}=0.24;\left[-1.26;1.67\right]$) did not seem to be related to density (Appendix S2: Figure S4).

## DISCUSSION

We show that OPSCR models can account for and estimate spatial variation in survival. Survival can be modeled as a function of any spatial covariate, including local density estimated within the same model. Using simulations, we show that the model produces sound inferences on the role of spatial covariates and density dependence in explaining spatial variation in survival. The model can also integrate spatial dead recoveries (Dupont et al., 2021) and estimate multiple competing sources of mortality with potentially different spatial determinants. The model is efficient and overcomes a challenge faced by other methods, namely, to obtain population‐level assessment of spatial determinants of variation in survival. The wolverine case study revealed country‐ and cause‐specific estimates of mortalities, highly relevant for the conservation and management of transboundary populations.

Despite the recognized potential of OPSCR models to estimate spatial variation in vital rates (Royle et al., 2014), few studies have attempted to use them for this purpose (Chandler et al., 2018). We are not aware of any study that evaluated the performance of OPSCR models that include spatially explicit vital rates. Our simulations showed that sound inferences in the spatial variation of cause‐specific mortality could be obtained when survival was modeled as a function of a deterministic spatial covariate, even with relatively small OPSCR data sets (5 occasions, ≈50 individuals detected per occasion). OPSCR model performance (convergence, precision) was significantly higher for simulations with spatially autocorrelated survival (gradient) compared with spatially random survival (Appendix S1: Tables S3–S6).

So far, it has only been possible to estimate the strength of density‐dependent survival within the same capture–recapture model using the overall density estimates (Schofield & Barker, 2008; Tenan et al., 2019). The approach described in this paper now makes it possible to estimate the strength of density‐dependent survival given the local density experienced by individuals. Although density is only a proxy to describe the interactions that occur between individuals, using local density is relevant to describe the conditions experienced by individuals within their home range. Simultaneous estimation of density and its link with survival within the same model propagates the uncertainty in both the number of individuals alive and their location to mortality estimates. Because density is both a latent variable and a covariate used to explain variation in survival, it is computationally challenging (i.e., due to the increased number of model dependencies) to fit density‐dependent survival OPSCR models. In Milleret (2022), we showcase how centering the density covariate (d) can improve the mixing of MCMC chains of different model parameters.

The lower survival of female wolverines in Norway linked with higher hunting mortality was expected given the Norwegian culling policy. However, in this study, mortality did not seem to be density‐dependent, in contrast to previous findings in this population (Brøseth et al., 2010), which could be because only the female part of the population was considered and because the OPSCR model estimates the effect of local density and not the effect of overall population density.

Future research should focus on building and testing models that estimate spatial variation in other demographic parameters, that is, recruitment, emigration, and immigration, because this is an essential step toward a complete understanding of the mechanisms driving spatial heterogeneity in density and, therefore spatial population dynamics (Chandler et al., 2018). In general, we lack methods for assessing violations of assumptions of Bayesian OPSCR models; this is a field that requires development (Dey et al., 2022). The Bayesian model written in NIMBLE and the set of features available in the nimbleSCR package allows users to fit efficient and flexible OPSCR models. This development represents an essential step toward a fully spatially explicit OPSCR model (Chandler et al., 2018).

## AUTHOR CONTRIBUTIONS

Cyril Milleret, Richard Bischof, Pierre Dupont, and Soumen Dey conceived and designed the study. Cyril Milleret implemented the analysis and wrote the manuscript with contributions from all authors. Henrik Brøseth provided the wolverine genetic data. All authors gave final approval for publication.

## CONFLICT OF INTEREST

The authors declare no conflict of interest.

## Supporting information


Appendix S1

Appendix S2

## Data Availability

Data and code (Milleret, 2022) to reproduce the analysis are available on Zenodo at https://doi.org/10.5281/zenodo.7267647.

## References

[ecy3934-bib-0001] Bischof, R. , C. Milleret , P. Dupont , J. Chipperfield , M. Tourani , A. Ordiz , P. de Valpine , et al. 2020. “Estimating and Forecasting Spatial Population Dynamics of Apex Predators Using Transnational Genetic Monitoring.” Proceedings of the National Academy of Sciences of the United States of America 117: 30531–8. 33199605 10.1073/pnas.2011383117 PMC7720137

[ecy3934-bib-0002] Bischof, R. , D. Turek , C. Milleret , T. Ergon , P. Dupont , and P. de Valpine . 2020. “nimbleSCR: Spatial Capture‐Recapture (SCR) Methods Using nimble.” https://cran.r-project.org/package=nimbleSCR.

[ecy3934-bib-0003] Bonenfant, C. , J.‐M. Gaillard , T. Coulson , M. Festa‐Bianchet , A. Loison , M. Garel , L. E. Loe , P. Blanchard , N. Pettorelli , and N. Owen‐Smith . 2009. “Empirical Evidence of Density‐Dependence in Populations of Large Herbivores.” Advances in Ecological Research 41: 313–57.

[ecy3934-bib-0004] Borchers, D. L. , and M. G. Efford . 2008. “Spatially Explicit Maximum Likelihood Methods for Capture‐Recapture Studies.” Biometrics 64: 377–85. 17970815 10.1111/j.1541-0420.2007.00927.x

[ecy3934-bib-0005] Brøseth, H. , Ø. Flagstad , C. Wärdig , M. Johansson , and H. Ellegren . 2010. “Large‐Scale Noninvasive Genetic Monitoring of Wolverines Using Scats Reveals Density Dependent Adult Survival.” Biological Conservation 143: 113–20.

[ecy3934-bib-0006] Chandler, R. B. , J. Hepinstall‐Cymerman , S. Merker , H. Abernathy‐Conners , and R. J. Cooper . 2018. “Characterizing Spatio‐Temporal Variation in Survival and Recruitment with Integrated Population Models.” The Auk 135: 409–26.

[ecy3934-bib-0007] de Valpine, P. , K. Scranton , J. Knape , K. Ram , and N. J. Mills . 2014. “The Importance of Individual Developmental Variation in Stage‐Structured Population Models.” Ecology Letters 17: 1026–38. 24811267 10.1111/ele.12290

[ecy3934-bib-0008] de Valpine, P. , D. Turek , C. J. Paciorek , C. Anderson‐Bergman , D. T. Lang , and R. Bodik . 2017. “Programming with Models: Writing Statistical Algorithms for General Model Structures with NIMBLE.” Journal of Computational and Graphical Statistics 26: 403–13.

[ecy3934-bib-0009] DeCesare, N. J. , M. Hebblewhite , M. Bradley , D. Hervieux , L. Neufeld , and M. Musiani . 2014. “Linking Habitat Selection and Predation Risk to Spatial Variation in Survival.” Journal of Animal Ecology 83: 343–52. 24099266 10.1111/1365-2656.12144 PMC4285818

[ecy3934-bib-0010] Dey, S. , R. Bischof , P. P. A. Dupont , and C. Milleret . 2022. “Does the Punishment Fit the Crime? Consequences and Diagnosis of Misspecified Detection Functions in Bayesian Spatial Capture‐Recapture Modeling.” Ecology and Evolution 12: e8600. 35222967 10.1002/ece3.8600 PMC8847120

[ecy3934-bib-0012] Dupont, P. , C. Milleret , M. Tourani , H. Brøseth , and R. Bischof . 2021. “Integrating Dead Recoveries in Open‐Population Spatial Capture‐Recapture Models.” Ecosphere 12: e03571.

[ecy3934-bib-0013] Efford, M. 2004. “Density Estimation in Live‐Trapping Studies.” Oikos 106: 598–610.

[ecy3934-bib-0034] Efford, M. G. , and M. R. Schofield . 2022. “A Review of Movement Models in Open Population Capture–recapture.” Methods in Ecology and Evolution 13: 2106–18. 10.1111/2041-210x.13947.

[ecy3934-bib-0014] Ergon, T. , Ø. Borgan , C. R. Nater , and Y. Vindenes . 2018. “The Utility of Mortality Hazard Rates in Population Analyses.” Methods in Ecology and Evolution 9: 2046–56.

[ecy3934-bib-0015] Gaillard, J.‐M. , M. Festa‐Bianchet , N. G. Yoccoz , A. Loison , and C. Toigo . 2000. “Temporal Variation in Fitness Components and Population Dynamics of Large Herbivores.” Annual Review of Ecology and Systematics 31: 367–93.

[ecy3934-bib-0016] Gardner, B. , R. Sollmann , N. S. Kumar , D. Jathanna , and K. U. Karanth . 2018. “State Space and Movement Specification in Open Population Spatial Capture‐Recapture Models.” Ecology and Evolution 8: 10336–44. 30397470 10.1002/ece3.4509 PMC6206188

[ecy3934-bib-0017] Gelman, A. , and D. B. Rubin . 1992. “Inference from Iterative Simulation Using Multiple Sequences.” Statistical Science 7: 457–511.

[ecy3934-bib-0018] Gimenez, O. , B. J. T. Morgan , and S. P. Brooks . 2009. “Weak Identifiability in Models for Mark‐Recapture‐Recovery Data.” In Modeling Demographic Processes in Marked Populations, edited by D. L. Thomson, E. G. Cooch, and M. J. Conroy, 1055–67. Boston, MA: Springer.

[ecy3934-bib-0019] Lebreton, J. D. , and R. Pradel . 2002. “Multistate Recapture Models: Modelling Incomplete Individual Histories.” Journal of Applied Statistics 29: 353–69.

[ecy3934-bib-0020] Milleret, C. 2022. “Cyril‐Milleret/OPSCRS Patial Survival Ecology: v1.0.6 (v.1.0.6).” Zenodo. 10.5281/zenodo.7267647.

[ecy3934-bib-0021] Milleret, C. , R. Bischof , P. Dupont , H. Brøseth , J. Odden , and J. Mattisson . 2021. “GPS Collars Have an Apparent Positive Effect on the Survival of a Large Carnivore.” Biology Letters 17: 20210128. 34186003 10.1098/rsbl.2021.0128 PMC8241484

[ecy3934-bib-0022] Milleret, C. , P. Dupont , C. Bonenfant , H. Brøseth , Ø. Flagstad , C. Sutherland , and R. Bischof . 2019. “A Local Evaluation of the Individual State‐Space to Scale Up Bayesian Spatial Capture‐Recapture.” Ecology and Evolution 9: 352–63. 30680119 10.1002/ece3.4751 PMC6342129

[ecy3934-bib-0023] Milleret, C. , P. Dupont , J. Chipperfield , D. Turek , H. Brøseth , O. Gimenez , P. de Valpine , and R. Bischof . 2020. “Estimating Abundance with Interruptions in Data Collection Using Open Population Spatial Capture‐Recapture Models.” Ecosphere 11: e03172.

[ecy3934-bib-0024] NIMBLE Development Team . 2019. “NIMBLE: MCMC, Particle Filtering, and Programmable Hierarchical Modeling.” https://cran.r-project.org/package=nimble.

[ecy3934-bib-0025] R Core Team . 2021. R: A Language and Environment for Statistical Computing. Vienna: R Foundation for Statistical Computing.

[ecy3934-bib-0026] Royle, J. A. , R. B. Chandler , R. Sollmann , and B. Gardner . 2014. Spatial Capture‐Recapture. Waltham, MA: Academic Press.

[ecy3934-bib-0027] Royle, J. A. , R. M. Dorazio , and W. A. Link . 2007. “Analysis of Multinomial Models with Unknown Index Using Data Augmentation.” Journal of Computational and Graphical Statistics 16(1): 67–85.

[ecy3934-bib-0028] Schofield, M. R. , and R. J. Barker . 2008. “A Unified Capture‐Recapture Framework.” Journal of Agricultural, Biological, and Environmental Statistics 13: 458–77.

[ecy3934-bib-0029] Sun, C. C. , A. K. Fuller , and J. A. Royle . 2014. “Trap Configuration and Spacing Influences Parameter Estimates in Spatial Capture‐Recapture Models.” PLoS One 9: 1–9. 10.1371/journal.pone.0088025 PMC3914876 24505361

[ecy3934-bib-0030] Tenan, S. , G. Tavecchia , D. Oro , and R. Pradel . 2019. “Assessing the Effect of Density on Population Growth when Modeling Individual Encounter Data.” Ecology 100: e02595. 30620394 10.1002/ecy.2595

[ecy3934-bib-0031] Turek, D. , C. Milleret , T. Ergon , H. Brøseth , P. Dupont , R. Bischof , and P. de Valpine . 2021. “Efficient Estimation of Large‐Scale Spatial Capture‐Recapture Models.” Ecosphere 12: e03385.

[ecy3934-bib-0032] Zhang, W. , J. D. Chipperfield , J. B. Illian , P. Dupont , C. Milleret , P. de Valpine , and R. Bischof . 2022. “A Flexible and Efficient Bayesian Implementation of Point Process Models for Spatial Capture–Recapture Data.” *Ecology* e3887. 10.1002/ecy.3887. PMC10078592 36217822

