# Supplementary material for: Estimating spatially variable and density‐dependent survival using open‐population spatial capture–recapture models
Source: Ecology. 2023 Jan 5;104(2):e3934. doi: 10.1002/ecy.3934 (PMC10078101; doi:10.1002/ecy.3934)
Supplement: Supplementary file 1 — Appendix S1 [file ECY-104-0-s001.pdf]

Appendix S1:
Estimating spatially variable and density-dependent survival using open-population spatial capture–recapture models

Cyril Milleret[1], Soumen Dey[1], Pierre Dupont[1], Henrik Brøseth[2], Daniel Turek[3], Perry de Valpine[4], Richard Bischof[1]

[1] Faculty of Environmental Sciences and Natural Resource Management, Norwegian University of Life Sciences, NO-1432 Ås, Norway.
[2] Norwegian Institute for Nature Research (NINA), NO-7485, Trondheim, Norway.
[3] Department of Mathematics and Statistics, Williams College, Williamstown, MA 01267, USA.
[4] Department of Environmental Science, Policy and Management, University of California Berkeley, Berkeley, USA.

Journal name: Ecology

1 Spatial heterogeneity in mortality using a deterministic spatial covariate

1.1 Accounting for spatial heterogeneity in mortality

Table S 1: Summary of the 16 simulation scenarios explored to explain heterogeneity in spatial mortality using a spatial covariate. The average number (and standard deviation), across all 100 datasets, of individuals considered alive ($z_i = 2$), detected alive, and recovered dead ($z_i = 3$) are shown for each of the 5 consecutive occasions.

N_1	β_h	β_w	**Spatial Covariate**	**Alive**					**Detected**					**Dead**				
				1	2	3	4	5	1	2	3	4	5	1	2	3	4	5
120	1	1	Gradient	119.6(9.2)	124.0(9.2)	125.4(9.7)	129.2(10.7)	133.3(9.5)	47.0(6.6)	50.5(6.5)	52.8(7.1)	55.6(7.3)	57.9(7.6)	0	12.3(3.3)	12.8(3.8)	12.7(3.2)	12.3(3.8)
120	-1	1	Gradient	250.7(11.3)	257.1(12.7)	264.0(14.1)	270.0(17.5)	275.3(17.3)	99.6(10.3)	105.8(9.7)	112.1(10.8)	117.7(12.2)	120.4(11.6)	0	25.7(5.4)	25.2(5.2)	26.6(5.0)	26.2(5.2)
120	1	-1	Gradient	121.2(10.0)	125.8(9.4)	126.3(9.3)	125.0(8.8)	127.0(9.8)	48.3(6.6)	51.9(5.9)	53.8(7.2)	54.2(7.8)	54.9(7.1)	0	12.5(3.4)	13.5(3.8)	13.6(4.2)	13.4(3.8)
120	-1	-1	Gradient	248.5(10.7)	254.3(11.4)	260.2(13.2)	261.4(12.8)	263.6(13.4)	98.0(8.4)	106.1(9.7)	111.5(9.6)	114.9(11.0)	116.0(10.3)	0	27.0(5.1)	26.6(5.7)	27.6(6.0)	28.2(6.1)
250	1	1	Gradient	120.0(10.2)	123.0(8.8)	124.2(8.6)	125.9(9.2)	128.2(11.0)	47.0(6.7)	49.9(6.8)	52.4(6.0)	54.2(7.2)	56.5(7.6)	0	12.7(4.1)	12.6(4.0)	13.1(3.6)	13.3(3.8)
250	-1	1	Gradient	248.0(10.9)	256.5(11.1)	263.8(13.0)	266.9(12.3)	266.4(14.0)	97.3(8.5)	107.2(9.0)	111.0(9.6)	116.1(8.7)	118.2(10.8)	0	26.3(5.9)	26.3(5.8)	28.2(5.3)	29.0(5.6)
250	1	-1	Gradient	119.6(8.0)	124.5(9.2)	128.3(8.7)	131.0(9.5)	133.2(11.5)	47.0(6.0)	51.8(6.8)	53.8(6.7)	56.8(7.0)	58.6(8.0)	0	11.6(3.4)	12.3(3.3)	12.7(3.7)	12.8(4.1)
250	-1	-1	Gradient	250.2(12.3)	258.9(12.6)	265.6(14.5)	271.6(17.5)	275.2(19.2)	99.3(9.6)	107.0(9.9)	112.0(10.4)	117.0(12.0)	120.5(14.3)	0	25.0(5.1)	25.9(5.4)	26.1(5.1)	27.1(6.0)
120	1	1	Random	119.8(8.8)	118.7(9.2)	123.4(8.1)	127.3(9.5)	134.4(9.2)	47.9(6.7)	51.2(6.4)	53.9(6.0)	56.3(6.7)	60.1(6.1)	0	14.2(3.4)	11.7(3.5)	12.2(3.1)	11.4(3.5)
120	-1	1	Random	249.1(11.5)	248.1(11.8)	257.5(12.5)	268.3(13.0)	281.6(12.6)	99.3(8.9)	108.1(9.0)	113.6(9.0)	119.8(9.9)	126.2(11.0)	0	29.1(4.6)	24.0(5.4)	25.4(4.8)	24.2(5.5)
120	1	-1	Random	120.0(8.9)	114.2(8.6)	112.8(8.0)	112.0(8.3)	112.0(8.5)	47.3(5.7)	48.4(6.7)	49.6(6.6)	51.1(6.9)	52.1(7.0)	0	17.0(3.9)	15.3(3.9)	15.0(3.7)	14.8(3.7)
120	-1	-1	Random	251.0(13.6)	241.5(12.2)	237.4(12.9)	234.4(12.3)	232.8(10.8)	100.1(8.8)	104.8(9.4)	107.7(9.5)	108.4(10.1)	109.4(9.4)	0	34.6(5.1)	31.3(5.1)	31.4(5.5)	31.5(5.5)
250	1	1	Random	120.7(8.7)	114.6(8.5)	112.9(8.8)	113.0(9.5)	113.3(9.7)	47.5(6.7)	48.9(6.2)	50.7(5.9)	52.0(6.3)	52.1(6.7)	0	16.4(3.8)	15.8(3.6)	15.9(4.4)	15.3(3.5)
250	-1	1	Random	250.5(11.6)	239.1(11.3)	236.9(12.5)	236.0(12.9)	235.5(12.3)	99.4(8.2)	102.2(8.9)	104.1(10.3)	105.8(8.7)	107.0(9.2)	0	33.2(5.2)	31.9(5.1)	31.5(5.8)	32.5(6.1)
250	1	-1	Random	119.9(8.7)	120.5(8.8)	126.0(10.6)	130.1(9.5)	137.1(7.8)	47.5(6.7)	49.7(6.8)	52.2(7.2)	55.6(6.8)	59.1(6.5)	0	13.5(3.4)	11.7(3.3)	11.7(3.2)	11.1(3.3)
250	-1	-1	Random	249.5(13.1)	248.9(12.4)	261.5(11.0)	273.7(12.0)	285.5(10.7)	99.5(10.2)	104.2(9.0)	111.3(9.0)	115.4(10.0)	120.4(10.0)	0	28.7(4.7)	23.4(4.1)	23.7(4.5)	24.1(4.7)

Table S 2: Percentage of models with all parameters converged, identifiable, and both converged and identifiable for the 100 datasets associated with each of the 16 scenarios tested.

N_1	β_h	β_w	Spatial Covariate	% Converged	% Identifiable	% Converged and Identifiable
120.00	1.00	1.00	Gradient	100	100	100
120.00	-1.00	1.00	Gradient	96	76	72
120.00	1.00	-1.00	Gradient	100	94	94
120.00	-1.00	-1.00	Gradient	100	100	100
250.00	1.00	1.00	Gradient	100	100	100
250.00	-1.00	1.00	Gradient	100	100	100
250.00	1.00	-1.00	Gradient	100	100	100
250.00	-1.00	-1.00	Gradient	100	100	100
120.00	1.00	1.00	Random	67	64	31
120.00	-1.00	1.00	Random	65	64	29
120.00	1.00	-1.00	Random	78	79	57
120.00	-1.00	-1.00	Random	57	71	28
250.00	1.00	1.00	Random	99	93	92
250.00	-1.00	1.00	Random	99	94	93
250.00	1.00	-1.00	Random	98	92	90
250.00	-1.00	-1.00	Random	99	93	92

Table S 3: Relative bias (RB) and coefficient of variation (CV) for the 100 simulated datasets for the scenario with β_h = 1, β_w = -1, and for a) N_1 = 120 and b) N_1 = 250. Values within brackets show the 95% quantiles.

(a) $N_1 = 120$

	**Random**			**Gradient**		
Parameters	RB	CV	Coverage	RB	CV	Coverage
$\beta_h = 1$ β_w = -1						
N_1	0.00 (-0.18-0.22)	10.29 (8.60-11.84)	93	0.02 (-0.20-0.18)	9.80 (8.69-11.33)	97
N_2	0.02 (-0.14-0.19)	8.74 (7.51-10.28)	97	0.03 (-0.13-0.16)	8.56 (7.63-9.79)	98
N_3	0.02 (-0.10-0.17)	8.42 (7.23-9.36)	97	0.04 (-0.09-0.21)	8.17 (7.24-9.04)	93
N_4	0.02 (-0.11-0.20)	8.42 (7.56-9.63)	91	0.04 (-0.08-0.15)	8.15 (7.33-9.08)	99
N_5	0.03 (-0.17-0.18)	9.38 (8.45-10.74)	96	0.05 (-0.09-0.19)	9.39 (8.44-10.94)	98
β_h	0.04 (-1.05-1.65)	55.69 (29.71-1414.65)	100	-0.01 (-0.41-0.49)	25.12 (19.90-36.51)	100
β_w	-0.06 (-1.80-1.54)	87.26 (46.80-913.09)	100	0.13 (-0.52-0.82)	33.68 (25.79-63.49)	100
τ	0.00 (-0.16-0.12)	7.24 (6.31-9.34)	100	-0.01 (-0.12-0.15)	7.33 (6.28-8.72)	100
mh_h	0.07 (-0.13-0.32)	10.51 (7.70-16.79)	100	0.02 (-0.17-0.22)	9.11 (8.15-10.61)	100
mh_w	0.04 (-0.24-0.52)	21.29 (14.89-35.62)	100	0.03 (-0.21-0.43)	16.68 (14.25-23.74)	100
p_0	0.01 (-0.13-0.15)	8.01 (7.34-9.14)	100	0.01 (-0.14-0.16)	8.10 (7.30-8.87)	100
σ	-0.01 (-0.07-0.08)	3.73 (3.39-4.38)	100	0.00 (-0.08-0.07)	3.75 (3.35-4.19)	100

(b) $N_1 = 250$

	**Random**			**Gradient**		
Parameters	RB	CV	Coverage	RB	CV	Coverage
$\beta_h = 1$ β_w = -1						
N_1	0.00 (-0.12-0.16)	7.14 (6.46-7.68)	94	0.01 (-0.10-0.14)	6.81 (6.40-7.45)	95
N_2	0.02 (-0.08-0.16)	6.00 (5.52-6.50)	94	0.01 (-0.08-0.12)	5.95 (5.56-6.40)	97
N_3	0.01 (-0.08-0.11)	5.65 (5.23-6.29)	97	0.01 (-0.11-0.12)	5.69 (5.24-6.32)	92
N_4	0.03 (-0.07-0.15)	5.66 (5.28-6.16)	95	0.02 (-0.08-0.14)	5.73 (5.21-6.12)	93
N_5	0.03 (-0.09-0.13)	6.32 (5.91-6.96)	97	0.01 (-0.10-0.14)	6.54 (6.01-7.16)	95
β_h	0.02 (-0.83-1.04)	40.95 (22.96-237.76)	100	0.00 (-0.31-0.37)	17.35 (14.73-22.84)	100
β_w	0.17 (-1.30-1.27)	55.96 (31.39-528.97)	100	-0.02 (-0.42-0.63)	23.56 (18.30-35.05)	100
τ	0.01 (-0.10-0.10)	4.92 (4.47-5.56)	100	-0.01 (-0.11-0.09)	5.12 (4.54-5.72)	100
mh_h	0.04 (-0.09-0.21)	7.08 (5.34-12.51)	100	0.00 (-0.08-0.15)	6.29 (5.69-6.86)	100
mh_w	0.08 (-0.18-0.39)	13.66 (9.96-22.01)	100	0.01 (-0.16-0.28)	10.90 (9.58-13.09)	100
p_0	0.01 (-0.07-0.11)	5.48 (5.19-5.88)	100	-0.02 (-0.10-0.14)	5.65 (5.28-6.12)	100
σ	-0.01 (-0.05-0.05)	2.55 (2.39-2.74)	100	0.00 (-0.06-0.05)	2.62 (2.42-2.89)	100

Table S 4: Relative bias (RB) and coefficient of variation (CV) for the 100 simulated datasets for the scenario with β_h = -1, β_w = -1, and for a) $N_1 = 120$ and b) $N_1 = 250$. Values within brackets show the 95% quantiles.

(a) $N_1 = 120$

	**Random**			**Gradient**		
Parameters	RB	CV	Coverage	RB	CV	Coverage
			β_h = -1 β_w = -1			
N_1	0.01 (-0.18-0.21)	10.22 (8.98-11.82)	96	0.03 (-0.17-0.20)	9.77 (8.54-11.37)	98
N_2	0.03 (-0.19-0.17)	8.62 (7.63-9.78)	91	0.02 (-0.15-0.18)	8.45 (7.47-9.62)	93
N_3	0.01 (-0.10-0.18)	8.18 (7.53-9.11)	96	0.03 (-0.13-0.16)	7.98 (7.06-9.46)	97
N_4	0.02 (-0.08-0.18)	8.29 (7.31-9.07)	95	0.03 (-0.11-0.18)	7.99 (7.01-9.30)	93
N_5	0.04 (-0.14-0.21)	9.27 (7.96-10.23)	96	0.05 (-0.16-0.20)	8.80 (7.77-10.09)	94
β_h	-0.03 (-1.20-1.93)	58.55 (33.61-881.07)	100	0.02 (-0.31-0.45)	22.45 (18.22-30.19)	100
β_w	0.07 (-1.64-1.51)	85.12 (46.30-735.50)	100	0.15 (-0.49-1.05)	32.91 (25.01-59.95)	100
τ	-0.02 (-0.15-0.10)	7.34 (6.09-8.93)	100	0.00 (-0.15-0.11)	7.24 (6.20-9.25)	100
mh_h	0.05 (-0.11-0.39)	9.18 (7.53-19.58)	100	0.01 (-0.13-0.17)	7.99 (7.27-8.93)	100
mh_w	0.08 (-0.17-0.55)	20.50 (14.98-34.06)	100	0.06 (-0.23-0.57)	16.46 (14.00-25.56)	100
p_0	0.02 (-0.13-0.15)	7.87 (7.14-8.79)	100	0.00 (-0.14-0.14)	7.93 (7.16-8.99)	100
σ	-0.01 (-0.07-0.06)	3.71 (3.35-4.15)	100	0.00 (-0.06-0.07)	3.72 (3.32-4.29)	100

(b) $N_1 = 250$

	**Random**			**Gradient**		
Parameters	RB	CV	Coverage	RB	CV	Coverage
			β_h = -1 β_w = -1			
N_1	0.02 (-0.13-0.15)	7.07 (6.49-7.87)	94	0.03 (-0.10-0.17)	6.95 (6.20-7.61)	91
N_2	0.01 (-0.11-0.14)	5.95 (5.40-6.59)	95	0.02 (-0.09-0.14)	5.87 (5.40-6.37)	93
N_3	0.01 (-0.10-0.10)	5.64 (5.32-6.31)	97	0.03 (-0.08-0.15)	5.54 (5.07-5.92)	91
N_4	0.01 (-0.10-0.12)	5.62 (5.09-6.29)	97	0.02 (-0.10-0.13)	5.52 (5.04-6.10)	90
N_5	0.01 (-0.12-0.15)	6.26 (5.65-7.10)	92	0.01 (-0.09-0.12)	6.13 (5.51-6.78)	96
β_h	0.13 (-0.73-1.07)	36.93 (22.78-138.11)	100	0.03 (-0.28-0.33)	15.49 (13.14-20.25)	100
β_w	0.11 (-1.09-1.57)	54.58 (32.03-612.74)	100	0.03 (-0.40-0.72)	22.55 (17.58-32.78)	100
τ	0.00 (-0.10-0.09)	5.01 (4.41-5.64)	100	0.00 (-0.09-0.10)	4.96 (4.43-5.63)	100
mh_h	0.02 (-0.08-0.19)	6.31 (5.12-10.94)	100	0.01 (-0.10-0.12)	5.51 (5.11-5.97)	100
mh_w	0.07 (-0.21-0.42)	13.23 (9.75-25.22)	100	0.02 (-0.20-0.35)	10.95 (9.82-13.29)	100
p_0	0.00 (-0.09-0.12)	5.46 (5.02-5.91)	100	-0.01 (-0.12-0.07)	5.53 (5.20-6.07)	100
σ	0.00 (-0.04-0.05)	2.57 (2.33-2.83)	100	0.00 (-0.05-0.05)	2.58 (2.40-2.84)	100

Table S 5: Relative bias (RB) and coefficient of variation (CV) for the 100 simulated datasets for the scenario with β_h = -1, β_w = 1, and for a) $N_1 = 120$ and b) $N_1 = 250$. Values within brackets show the 95% quantiles.

(a) $N_1 = 120$

	**Random**			**Gradient**		
Parameters	RB	CV	Coverage	RB	CV	Coverage
			β_h = -1 $\beta_w = 1$			
N_1	0.04 (-0.12-0.16)	9.91 (8.80-11.69)	100	0.03 (-0.18-0.14)	9.88 (8.79-11.28)	95
N_2	0.03 (-0.10-0.17)	8.41 (7.50-9.39)	96	0.02 (-0.11-0.21)	8.53 (7.55-9.94)	95
N_3	0.05 (-0.18-0.17)	8.15 (7.12-9.52)	93	0.02 (-0.16-0.15)	8.33 (7.41-9.16)	95
N_4	0.02 (-0.13-0.19)	8.25 (7.35-9.45)	95	0.01 (-0.12-0.17)	8.30 (7.64-9.71)	98
N_5	0.02 (-0.19-0.20)	9.36 (8.54-10.65)	93	0.04 (-0.17-0.21)	9.65 (8.39-10.79)	96
β_h	0.09 (-0.82-1.35)	57.05 (32.58-271.78)	100	0.01 (-0.37-0.60)	20.94 (17.58-30.28)	100
β_w	0.44 (-1.20-2.26)	69.30 (37.07-440.05)	100	0.15 (-0.72-1.72)	40.41 (29.57-122.99)	100
τ	-0.01 (-0.15-0.15)	7.18 (6.36-8.31)	100	0.00 (-0.15-0.13)	7.39 (6.47-8.49)	100
mh_h	0.04 (-0.07-0.34)	10.80 (7.71-18.84)	100	0.04 (-0.15-0.23)	8.80 (7.89-10.88)	100
mh_w	0.09 (-0.22-0.61)	22.13 (15.16-37.25)	100	0.03 (-0.25-0.66)	16.92 (13.76-38.40)	100
p_0	-0.01 (-0.15-0.13)	7.93 (7.23-8.67)	100	0.00 (-0.16-0.20)	8.07 (7.34-9.05)	100
σ	0.01 (-0.06-0.07)	3.75 (3.33-4.17)	100	-0.01 (-0.08-0.08)	3.77 (3.41-4.20)	100

(b) $N_1 = 250$

	**Random**			**Gradient**		
Parameters	RB	CV	Coverage	RB	CV	Coverage
			β_h = -1 $\beta_w = 1$			
N_1	0.02 (-0.10-0.13)	7.03 (6.56-7.79)	98	0.03 (-0.13-0.14)	6.78 (6.16-7.39)	94
N_2	0.03 (-0.13-0.14)	6.01 (5.57-6.62)	90	0.01 (-0.09-0.13)	5.87 (5.39-6.40)	93
N_3	0.03 (-0.10-0.13)	5.70 (5.18-6.29)	96	0.03 (-0.09-0.12)	5.66 (5.17-6.10)	96
N_4	0.03 (-0.09-0.14)	5.70 (5.20-6.32)	91	0.02 (-0.10-0.13)	5.71 (5.20-6.20)	93
N_5	0.02 (-0.12-0.15)	6.43 (5.91-7.01)	90	0.02 (-0.08-0.14)	6.53 (5.98-7.07)	95
β_h	0.04 (-0.57-0.82)	39.67 (24.41-96.64)	100	-0.02 (-0.23-0.37)	14.47 (12.23-17.78)	100
β_w	0.02 (-1.01-1.62)	52.19 (31.31-471.12)	100	0.08 (-0.46-0.71)	25.28 (20.93-46.17)	100
τ	0.01 (-0.10-0.11)	5.02 (4.50-5.74)	100	-0.01 (-0.10-0.09)	4.99 (4.47-5.69)	100
mh_h	0.03 (-0.11-0.23)	7.14 (5.45-11.84)	100	0.02 (-0.12-0.16)	5.99 (5.49-6.52)	100
mh_w	0.03 (-0.18-0.48)	14.53 (10.09-25.79)	100	0.03 (-0.15-0.27)	10.64 (9.17-13.70)	100
p_0	-0.01 (-0.10-0.10)	5.52 (5.15-6.09)	100	0.00 (-0.10-0.11)	5.57 (5.09-5.96)	100
σ	0.00 (-0.04-0.05)	2.60 (2.38-2.90)	100	0.00 (-0.05-0.04)	2.58 (2.32-2.83)	100

Table S 6: Relative bias (RB) and coefficient of variation (CV) for the 100 simulated datasets for the scenario with $\beta_h = 1$, $\beta_w = 1$, and for a) $N_1 = 120$ and b) $N_1 = 250$. Values within brackets show the 95% quantiles.

(a) $N_1 = 120$

	**Random**			**Gradient**		
Parameters	RB	CV	Coverage	RB	CV	Coverage
			$\beta_h = 1$ $\beta_w = 1$			
N_1	0.01 (-0.13-0.18)	10.26 (8.91-12.14)	97	0.03 (-0.14-0.28)	9.93 (8.54-11.10)	90
N_2	0.01 (-0.18-0.16)	8.66 (7.56-9.94)	95	0.01 (-0.13-0.16)	8.36 (7.17-9.66)	97
N_3	0.02 (-0.12-0.19)	8.20 (7.38-9.37)	94	0.01 (-0.13-0.15)	7.83 (6.86-8.76)	95
N_4	0.01 (-0.15-0.14)	8.29 (7.31-9.49)	98	0.01 (-0.11-0.15)	7.73 (6.94-8.79)	99
N_5	0.00 (-0.14-0.18)	9.24 (8.17-10.02)	98	0.02 (-0.11-0.18)	8.44 (7.60-9.56)	96
β_h	-0.04 (-1.31-1.47)	61.72 (33.12-565.20)	100	0.05 (-0.44-0.60)	24.15 (19.24-38.92)	100
β_w	0.18 (-1.03-1.65)	83.17 (50.00-4032.25)	100	0.10 (-0.68-1.19)	38.86 (25.78-114.36)	100
τ	0.01 (-0.13-0.14)	7.22 (6.42-8.39)	100	0.01 (-0.11-0.13)	6.98 (6.03-8.05)	100
mh_h	0.05 (-0.12-0.30)	9.76 (7.61-18.42)	100	0.02 (-0.13-0.16)	8.23 (7.28-9.45)	100
mh_w	0.10 (-0.23-0.55)	22.37 (14.28-38.08)	100	0.07 (-0.20-0.39)	16.16 (13.30-23.20)	100
p_0	0.00 (-0.13-0.14)	7.98 (7.33-8.96)	100	0.01 (-0.13-0.16)	7.76 (7.10-8.59)	100
σ	0.00 (-0.07-0.09)	3.75 (3.39-4.23)	100	0.00 (-0.07-0.06)	3.63 (3.24-4.15)	100

(b) $N_1 = 250$

	**Random**			**Gradient**		
Parameters	RB	CV	Coverage	RB	CV	Coverage
			$\beta_h = 1$ $\beta_w = 1$			
N_1	0.02 (-0.11-0.16)	7.02 (6.48-7.83)	93	0.02 (-0.10-0.20)	6.90 (6.26-7.46)	92
N_2	0.00 (-0.10-0.15)	5.96 (5.50-6.56)	94	0.03 (-0.07-0.14)	5.81 (5.37-6.28)	96
N_3	0.02 (-0.08-0.12)	5.64 (5.22-6.27)	96	0.02 (-0.10-0.12)	5.37 (4.94-5.72)	93
N_4	0.02 (-0.08-0.15)	5.67 (5.07-6.21)	93	0.02 (-0.09-0.14)	5.25 (4.85-5.75)	91
N_5	0.02 (-0.13-0.15)	6.25 (5.70-6.92)	92	0.01 (-0.15-0.13)	5.75 (5.14-6.32)	89
β_h	0.17 (-0.59-1.13)	35.16 (24.21-112.59)	100	0.00 (-0.32-0.42)	17.00 (14.44-21.29)	100
β_w	0.14 (-1.02-1.50)	51.53 (32.64-740.10)	100	0.06 (-0.55-1.00)	25.00 (18.37-48.81)	100
τ	0.00 (-0.09-0.10)	5.00 (4.43-5.49)	100	-0.01 (-0.09-0.08)	4.83 (4.44-5.41)	100
mh_h	0.04 (-0.08-0.32)	6.88 (5.28-12.92)	100	0.01 (-0.10-0.12)	5.61 (5.26-6.12)	100
mh_w	0.05 (-0.16-0.51)	14.89 (9.44-26.22)	100	0.01 (-0.15-0.36)	10.44 (9.43-14.20)	100
p_0	0.00 (-0.10-0.12)	5.45 (5.06-6.00)	100	0.01 (-0.10-0.09)	5.40 (5.08-5.71)	100
σ	0.00 (-0.05-0.05)	2.55 (2.36-2.84)	100	0.00 (-0.04-0.05)	2.50 (2.34-2.69)	100

1.2 Not accounting for spatial heterogeneity in mortality

Table S 7: Percentage of models converged, identifiable and, both converged and identifiable for the 100 simulated datasets of 16 scenarios tested when misspecifying the OPSCR model by not accounting for spatial heterogeneity in mortality.

N_1	β_h	β_w	Spatial Covariate	% Converged	% Identifiable	% Converged and Identifiable
120.00	1.00	1.00	Gradient	100	99	99
120.00	-1.00	1.00	Gradient	100	100	100
120.00	1.00	-1.00	Gradient	100	100	100
120.00	-1.00	-1.00	Gradient	100	100	100
250.00	1.00	1.00	Gradient	100	100	100
250.00	-1.00	1.00	Gradient	100	100	100
250.00	1.00	-1.00	Gradient	100	100	100
250.00	-1.00	-1.00	Gradient	100	100	100
120.00	1.00	1.00	Random	100	99	99
120.00	-1.00	1.00	Random	100	99	99
120.00	1.00	-1.00	Random	100	100	100
120.00	-1.00	-1.00	Random	100	98	98
250.00	1.00	1.00	Random	100	100	100
250.00	-1.00	1.00	Random	100	100	100
250.00	1.00	-1.00	Random	100	100	100
250.00	-1.00	-1.00	Random	100	100	100

Table S 8: Relative bias (RB) and coefficient of variation (CV) for the 100 simulated datasets of the scenario with $\beta_h = 1$, $\beta_w = -1$, and a) $N_1 = 120$ and b) $N_1 = 250$ when not accounting for spatial heterogeneity in mortality. * denotes parameters for which we reported the average parameter value instead of RB, as RB could not be computed due to model misspecification. Values within brackets show the 95% quantiles.

(a) $N_1 = 120$

	**Random**			**Gradient**		
Parameters	RB	CV	Coverage	RB	CV	Coverage
			$\beta_h = 1$ $\beta_w = -1$			
N_1	0.02 (-0.18-0.20)	10.31 (8.67-11.83)	95	0.07 (-0.14-0.24)	9.82 (8.31-11.39)	91
N_2	0.02 (-0.13-0.20)	8.74 (7.55-10.13)	95	0.10 (-0.08-0.23)	8.53 (7.66-9.64)	88
N_3	0.02 (-0.10-0.19)	8.30 (7.28-9.30)	94	0.11 (-0.05-0.27)	8.26 (7.31-9.13)	80
N_4	0.02 (-0.11-0.20)	8.35 (7.55-9.40)	93	0.10 (-0.04-0.21)	8.29 (7.48-9.25)	91
N_5	0.03 (-0.16-0.23)	9.27 (8.33-10.63)	94	0.08 (-0.08-0.23)	9.55 (8.67-10.90)	94
β_h	-	-	-	-	-	-
β_w	-	-	-	-	-	-
τ	-0.01 (-0.16-0.11)	7.32 (6.38-9.37)	94	-0.02 (-0.14-0.14)	7.36 (6.39-8.86)	96
mh_h*	-2.13 (-2.46–1.78)	7.38 (6.82-8.50)	-	-1.90 (-2.16–1.65)	7.68 (7.00-8.65)	-
mh_w*	-1.65 (-2.17–1.14)	13.69 (12.25-16.53)	-	-1.56 (-2.00–1.24)	13.43 (12.27-15.07)	-
p_0	0.01 (-0.13-0.15)	7.97 (7.35-8.99)	97	0.01 (-0.15-0.15)	8.08 (7.27-8.86)	98
σ	-0.01 (-0.07-0.07)	3.72 (3.36-4.31)	95	0.00 (-0.08-0.07)	3.73 (3.35-4.17)	95

(b) $N_1 = 250$

	**Random**			**Gradient**		
Parameters	RB	CV	Coverage	RB	CV	Coverage
			$\beta_h = 1$ $\beta_w = -1$			
N_1	0.01 (-0.11-0.17)	7.09 (6.45-7.68)	93	0.06 (-0.07-0.18)	6.82 (6.33-7.44)	88
N_2	0.02 (-0.08-0.16)	6.00 (5.50-6.46)	94	0.07 (-0.02-0.18)	5.96 (5.53-6.47)	76
N_3	0.00 (-0.09-0.12)	5.62 (5.25-6.30)	94	0.07 (-0.05-0.19)	5.74 (5.35-6.28)	74
N_4	0.03 (-0.07-0.13)	5.65 (5.28-6.17)	96	0.07 (-0.03-0.19)	5.79 (5.28-6.30)	75
N_5	0.02 (-0.11-0.13)	6.36 (5.91-6.95)	98	0.05 (-0.08-0.19)	6.68 (6.11-7.26)	87
β_h	-	-	-	-	-	-
β_w	-	-	-	-	-	-
τ	0.00 (-0.09-0.10)	4.97 (4.39-5.67)	94	-0.02 (-0.12-0.08)	5.12 (4.57-5.79)	93
mh_h*	-2.12 (-2.37–1.85)	5.11 (4.82-5.58)	-	-1.88 (-2.12–1.74)	5.34 (5.00-5.77)	-
mh_w*	-1.61 (-2.04–1.29)	9.22 (8.55-9.93)	-	-1.51 (-1.83–1.30)	9.22 (8.57-10.01)	-
p_0	0.01 (-0.07-0.12)	5.50 (5.15-5.93)	95	-0.02 (-0.10-0.14)	5.63 (5.27-6.13)	93
σ	-0.01 (-0.05-0.05)	2.55 (2.39-2.77)	94	0.00 (-0.06-0.05)	2.61 (2.39-2.85)	89

Table S 9: Relative bias (RB) and coefficient of variation (CV) for the 100 simulated datasets of the scenario with β_h = -1, β_w = -1, and a) N_1 = 120 and b) N_1 = 250 when not accounting for spatial heterogeneity in mortality. * denotes parameters for which we reported the average parameter value instead of RB, as RB could not be computed due to model misspecification. Values within brackets show the 95% quantiles.

(a) $N_1 = 120$

	Random			Gradient		
Parameters	RB	CV	Coverage	RB	CV	Coverage
			β_h = -1 β_w = -1			
N_1	0.03 (-0.15-0.20)	10.21 (8.78-11.59)	98	0.04 (-0.15-0.22)	9.94 (8.49-11.71)	95
N_2	0.04 (-0.18-0.20)	8.54 (7.64-10.12)	92	0.02 (-0.15-0.21)	8.75 (7.67-9.84)	94
N_3	0.02 (-0.12-0.20)	8.15 (7.23-9.18)	92	0.02 (-0.16-0.14)	8.33 (7.38-9.76)	97
N_4	0.02 (-0.13-0.18)	8.20 (7.17-9.30)	94	-0.01 (-0.16-0.16)	8.22 (7.50-9.35)	95
N_5	0.04 (-0.17-0.20)	9.03 (8.05-10.23)	94	-0.01 (-0.20-0.16)	9.03 (8.24-10.03)	95
β_h	-	-	-	-	-	-
β_w	-	-	-	-	-	-
τ	-0.01 (-0.18-0.17)	7.11 (6.25-8.80)	88	0.00 (-0.15-0.12)	7.21 (6.20-9.05)	95
mh_h*	-2.22 (-2.55–1.89)	7.25 (6.69-8.23)	-	-2.20 (-2.51–1.91)	7.34 (6.76-8.32)	-
mh_w*	-1.70 (-2.23–1.27)	13.65 (12.27-16.21)	-	-1.65 (-2.13–1.24)	13.71 (12.02-15.69)	-
p_0	0.01 (-0.14-0.14)	7.84 (7.16-8.78)	96	0.00 (-0.14-0.15)	7.89 (7.21-8.86)	97
σ	-0.01 (-0.07-0.06)	3.68 (3.33-4.14)	96	0.01 (-0.06-0.07)	3.75 (3.32-4.25)	96

(b) $N_1 = 250$

	Random			Gradient		
Parameters	RB	CV	Coverage	RB	CV	Coverage
			β_h = -1 β_w = -1			
N_1	0.02 (-0.13-0.15)	7.05 (6.48-7.88)	94	0.04 (-0.09-0.22)	7.02 (6.21-7.64)	88
N_2	0.01 (-0.12-0.13)	5.97 (5.47-6.56)	93	0.03 (-0.08-0.14)	6.09 (5.65-6.67)	94
N_3	0.01 (-0.11-0.11)	5.64 (5.25-6.27)	95	0.01 (-0.09-0.15)	5.76 (5.35-6.21)	93
N_4	0.01 (-0.10-0.12)	5.59 (5.09-6.17)	94	-0.02 (-0.14-0.11)	5.72 (5.25-6.23)	90
N_5	0.00 (-0.11-0.15)	6.27 (5.63-6.95)	92	-0.04 (-0.14-0.08)	6.26 (5.77-6.83)	94
β_h	-	-	-	-	-	-
β_w	-	-	-	-	-	-
τ	-0.01 (-0.10-0.09)	4.97 (4.42-5.84)	94	0.01 (-0.09-0.11)	5.04 (4.55-6.02)	92
mh_h*	-2.19 (-2.43–1.91)	5.05 (4.73-5.54)	-	-2.20 (-2.42–1.98)	5.10 (4.81-5.50)	-
mh_w*	-1.65 (-2.04–1.29)	9.24 (8.56-9.99)	-	-1.62 (-1.97–1.32)	9.28 (8.58-9.94)	-
p_0	0.00 (-0.09-0.13)	5.47 (5.02-5.87)	95	-0.01 (-0.12-0.07)	5.53 (5.20-6.05)	91
σ	0.00 (-0.04-0.05)	2.57 (2.33-2.78)	98	0.00 (-0.05-0.05)	2.59 (2.41-2.85)	93

Table S 10: Relative bias (RB) and coefficient of variation (CV) for the 100 simulated datasets of the scenario with β_h = -1, β_w = 1, and a) N_1 = 120 and b) N_1 = 250 when not accounting for spatial heterogeneity in mortality. * denotes parameters for which we reported the average parameter value instead of RB, as RB could not be computed due to model misspecification. Values within brackets show the 95% quantiles.

(a) $N_1 = 120$

	Random			Gradient		
Parameters	RB	CV	Coverage	RB	CV	Coverage
			β_h = -1 $\beta_w = 1$			
N_1	0.04 (-0.14-0.18)	10.10 (8.81-11.77)	100	0.04 (-0.12-0.17)	9.75 (8.59-10.93)	99
N_2	0.03 (-0.10-0.16)	8.55 (7.48-9.60)	98	0.06 (-0.08-0.22)	8.55 (7.40-9.92)	92
N_3	0.04 (-0.15-0.16)	8.15 (7.18-9.38)	95	0.07 (-0.10-0.20)	8.31 (7.37-9.24)	96
N_4	0.03 (-0.13-0.20)	8.34 (7.17-9.51)	95	0.07 (-0.10-0.21)	8.47 (7.51-9.47)	89
N_5	0.01 (-0.19-0.20)	9.28 (8.50-10.68)	94	0.08 (-0.13-0.27)	9.63 (8.50-10.74)	91
β_h	-	-	-	-	-	-
β_w	-	-	-	-	-	-
τ	0.00 (-0.15-0.13)	7.17 (6.39-8.61)	95	-0.01 (-0.16-0.13)	7.32 (6.19-8.78)	91
mh_h*	-2.12 (-2.52–1.81)	7.37 (6.83-8.32)	-	-1.91 (-2.11–1.63)	7.77 (7.09-8.90)	-
mh_w*	-1.61 (-2.13–1.18)	13.66 (12.25-15.56)	-	-1.65 (-2.22–1.25)	13.81 (12.39-16.39)	-
p_0	-0.02 (-0.12-0.13)	7.98 (7.24-9.04)	98	0.00 (-0.16-0.19)	8.12 (7.27-8.97)	92
σ	0.01 (-0.06-0.07)	3.76 (3.36-4.27)	97	0.00 (-0.08-0.08)	3.78 (3.30-4.25)	94

(b) $N_1 = 250$

	Random			Gradient		
Parameters	RB	CV	Coverage	RB	CV	Coverage
			β_h = -1 $\beta_w = 1$			
N_1	0.03 (-0.10-0.14)	7.04 (6.57-7.76)	98	0.04 (-0.10-0.16)	6.72 (6.12-7.32)	91
N_2	0.03 (-0.11-0.16)	6.02 (5.48-6.78)	93	0.05 (-0.05-0.17)	5.84 (5.28-6.40)	87
N_3	0.03 (-0.09-0.13)	5.64 (5.20-6.29)	93	0.07 (-0.05-0.17)	5.66 (5.09-6.03)	83
N_4	0.02 (-0.08-0.14)	5.65 (5.26-6.31)	91	0.07 (-0.05-0.19)	5.74 (5.22-6.21)	83
N_5	0.02 (-0.11-0.15)	6.39 (5.78-6.94)	93	0.06 (-0.04-0.17)	6.53 (6.13-7.07)	84
β_h	-	-	-	-	-	-
β_w	-	-	-	-	-	-
τ	0.00 (-0.11-0.10)	5.04 (4.39-5.83)	91	-0.01 (-0.10-0.09)	5.03 (4.44-5.79)	94
mh_h*	-2.11 (-2.43–1.82)	5.15 (4.85-5.56)	-	-1.91 (-2.08–1.69)	5.33 (4.95-5.84)	-
mh_w*	-1.60 (-2.06–1.27)	9.25 (8.53-10.16)	-	-1.67 (-1.97–1.42)	9.28 (8.55-10.04)	-
p_0	-0.02 (-0.10-0.10)	5.52 (5.12-6.14)	96	0.00 (-0.10-0.11)	5.54 (5.07-5.93)	94
σ	0.00 (-0.05-0.05)	2.59 (2.37-2.88)	96	0.00 (-0.05-0.04)	2.57 (2.35-2.81)	98

Table S 11: Relative bias (RB) and coefficient of variation (CV) for the 100 simulated datasets of the scenario with $\beta_h = 1$, $\beta_w = 1$, and a) $N_1 = 120$ and b) $N_1 = 250$ when not accounting for spatial heterogeneity in mortality. * denotes parameters for which we reported the average parameter value (95% quantiles) instead of RB, as RB could not be computed due to model misspecification. Values within brackets show the 95% quantiles.

(a) $N_1 = 120$

	**Random**			**Gradient**		
Parameters	RB	CV	Coverage	RB	CV	Coverage
			$\beta_h = 1$ $\beta_w = 1$			
N_1	0.02 (-0.15-0.20)	10.22 (8.83-12.21)	98	0.07 (-0.10-0.32)	9.90 (8.33-10.95)	86
N_2	0.02 (-0.14-0.17)	8.61 (7.66-9.89)	95	0.06 (-0.07-0.21)	8.47 (7.21-9.73)	93
N_3	0.03 (-0.13-0.19)	8.30 (7.28-9.31)	95	0.04 (-0.09-0.19)	8.06 (7.18-9.06)	93
N_4	0.01 (-0.15-0.20)	8.33 (7.32-9.38)	91	0.03 (-0.11-0.19)	8.07 (7.32-9.07)	95
N_5	0.01 (-0.15-0.19)	9.20 (8.13-10.07)	96	0.02 (-0.11-0.18)	8.92 (8.21-9.86)	98
β_h	-	-	-	-	-	-
β_w	-	-	-	-	-	-
τ	0.00 (-0.13-0.13)	7.09 (6.15-8.71)	97	0.00 (-0.12-0.13)	7.00 (6.11-8.16)	98
mh_h*	-2.17 (-2.49–1.84)	7.39 (6.72-8.25)	-	-2.23 (-2.54–1.90)	7.20 (6.67-7.94)	-
mh_w*	-1.66 (-2.26–1.27)	13.73 (12.43-16.53)	-	-1.81 (-2.19–1.44)	13.50 (12.19-16.26)	-
p_0	0.00 (-0.14-0.14)	8.01 (7.16-8.84)	98	0.01 (-0.13-0.17)	7.76 (7.15-8.62)	95
σ	-0.01 (-0.07-0.08)	3.76 (3.31-4.18)	94	0.00 (-0.07-0.06)	3.62 (3.25-4.15)	94

(b) $N_1 = 250$

	**Random**			**Gradient**		
Parameters	RB	CV	Coverage	RB	CV	Coverage
			$\beta_h = 1$ $\beta_w = 1$			
N_1	0.03 (-0.12-0.16)	7.02 (6.35-7.85)	95	0.06 (-0.06-0.23)	6.90 (6.33-7.43)	87
N_2	0.00 (-0.09-0.15)	5.97 (5.46-6.59)	94	0.07 (-0.02-0.18)	5.90 (5.47-6.36)	75
N_3	0.01 (-0.09-0.13)	5.65 (5.25-6.31)	94	0.05 (-0.09-0.16)	5.58 (5.17-5.97)	82
N_4	0.02 (-0.08-0.14)	5.67 (5.09-6.21)	95	0.04 (-0.07-0.19)	5.57 (5.18-6.00)	89
N_5	0.01 (-0.14-0.15)	6.25 (5.79-6.89)	93	0.01 (-0.14-0.16)	6.10 (5.65-6.63)	88
β_h	-	-	-	-	-	-
β_w	-	-	-	-	-	-
τ	0.00 (-0.09-0.10)	4.97 (4.43-5.67)	95	-0.01 (-0.09-0.08)	4.81 (4.43-5.33)	97
mh_h*	-2.17 (-2.45–1.93)	5.08 (4.57-5.50)	-	-2.23 (-2.42–2.03)	4.96 (4.68-5.30)	-
mh_w*	-1.62 (-2.09-1.36)	9.25 (8.48-10.18)	-	-1.79 (-2.22–1.53)	9.08 (8.57-9.76)	-
p_0	0.01 (-0.10-0.12)	5.46 (5.01-5.95)	94	0.00 (-0.10-0.10)	5.40 (5.08-5.70)	97
σ	0.00 (-0.05-0.05)	2.56 (2.34-2.79)	92	0.00 (-0.04-0.05)	2.52 (2.34-2.68)	95

2 Density dependence in survival

Table S 12: Summary of the simulation with an effect of local density on survival. The average number (and standard deviation), across all 100 datasets, of individuals considered alive ($z_i = 2$), and detected alive for all consecutive occasions.

N_1	β_{phi}	β_{phi_0}	**Alive**					**Detected**				
N_1	β_{phi}	β_{phi_0}	1	2	3	4	5	1	2	3	4	5
250	-1	1.6	253.2(11.6)	223.8(10.4)	213.0(10.4)	207.9(11.2)	206.0(10.6)	149.4(10.8)	136.5(10.3)	131.2(8.8)	129.9(9.9)	131.2(9.5)

Table S 13: Percentage of models converged, identifiable and both converged and identifiable for the 100 simulated datasets. a)"With" and b)"Without" represent results obtained when fitting an OPSCR model that accounted and that did not account for the effect of density on survival, respectively. Note that both models were fitted to the same 100 replicated datasets.

(a) *With*

N_1	β_{phi}	β_{phi_0}	% Converged	% Identifiable	% Converged and Identifiable
250.00	-1.00	1.60	100	86	86

(b) *Without*

[redacted]	β_{phi}	β_{phi_0}	% Converged	% Identifiable	% Converged and Identifiable
250.00	-1.00	1.60	100	100	100

Table S 14: Relative bias (RB), standard deviation (SD) and coverage for the 100 replicated datasets of the local effect of density on survival. "With" and "Without" represent results obtained when fitting an OPSCR model that accounted and that did not account for the effect of density on survival, respectively. Note that the same 100 data sets was fitted to both models. * Indicates estimates for which RB could not be computed because the model was misspecified. Values within brackets show the 95% quantiles.

[redacted]	**With**			**Without**		
Parameters	RB	SD	Coverage	RB	SD	Coverage
N_1	0.01 (-0.10-0.11)	13.49 (12.43-14.64)	92	0.01 (-0.10-0.12)	13.19 (12.16-14.61)	92
N_2	0.00 (-0.08-0.08)	10.45 (9.61-11.44)	99	0.01 (-0.06-0.10)	10.60 (9.84-11.51)	97
N_3	0.00 (-0.08-0.09)	9.81 (8.92-10.64)	97	0.00 (-0.06-0.09)	9.90 (9.07-10.68)	96
N_4	0.00 (-0.06-0.09)	9.73 (8.87-10.81)	97	0.00 (-0.07-0.09)	9.78 (8.99-10.71)	98
N_5	0.01 (-0.06-0.12)	10.09 (9.12-11.06)	92	0.00 (-0.07-0.11)	10.19 (9.33-11.35)	96
β_ϕ	0.13 (-0.63-0.82)	0.43 (0.32-0.61)	96	-	-	-
τ	0.00 (-0.08-0.06)	0.03 (0.02-0.03)	98	0.00 (-0.08-0.06)	0.03 (0.02-0.03)	98
p_0	0.00 (-0.10-0.07)	0.00 (0.00-0.00)	94	0.00 (-0.10-0.07)	0.00 (0.00-0.01)	94
ϕ_0	0.13 (-0.70-0.83)	0.73 (0.53-1.04)	96	-0.05 (-0.17-0.05)*	0.06 (0.06-0.06)	-
σ	0.00 (-0.03-0.05)	0.01 (0.01-0.01)	98	0.00 (-0.03-0.05)	0.01 (0.01-0.01)	96

3 Mapping spatial heterogeneity in mortality

We quantified the overall deviation between simulated and OPSCR model predicted spatial maps of mortality. With Bayesian models, we can plot 1) the predicted spatial pattern of w and h (using eqn (3) and (4) in the main text), but also 2) the realized w and h for each habitat cell using the individual posterior z and s locations. We computed the average predictions of w and h for both methods for each cell r of the spatial domain S and then computed the cell by cell relative error between the simulated and predicted maps. Cell relative error was averaged to obtain a single measure of overall relative error for each replicated dataset. Because we did not consider time-dependence in mortality, we only obtained one relative error measure when using the predicted spatial pattern from w and h (1)). However, we computed the relative error for each transition (4 in total) for the realized w and h (2)) since we had access to all four survival transitions using the individual posterior z and s. We performed the calculation described above only for two scenarios with small ($N_1 = 120$) and large ($N_1 = 250$) population size, with a gradient deterministic spatial covariate, $\beta_w = 1$ and $\beta_h = -1$. For computational reasons, we thinned z and s by 10, which resulted in a total of 8400 posterior samples (i.e. 2800 samples per chain).

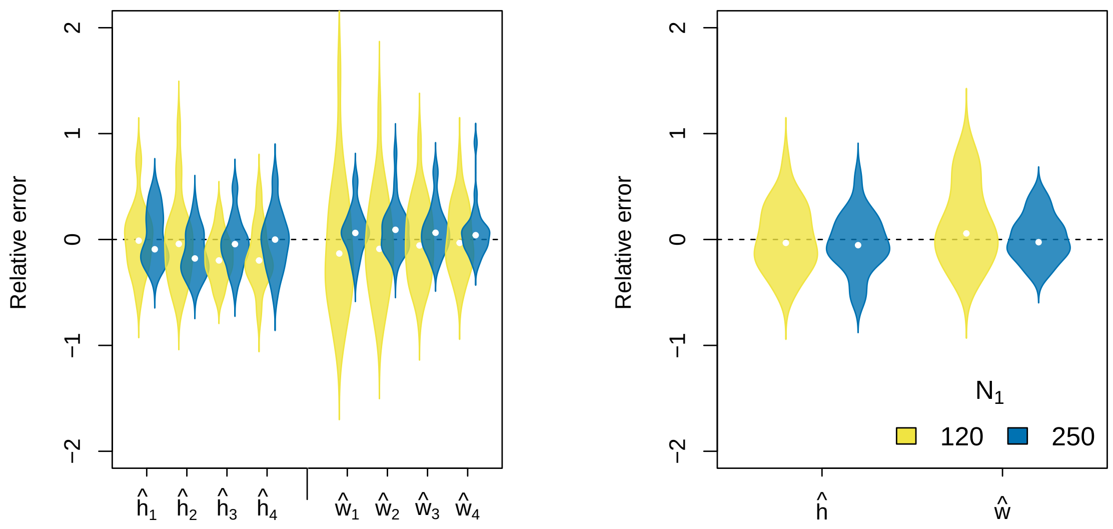

Figure S 1: Violin plots of the distribution of the relative error in the spatial pattern of cause-specific mortality probabilities ($\hat{h}$ and $\hat{w}$) obtained for each transition from the posterior s and z (left) and the predicted $\hat{h}$ and $\hat{w}$ (right). Results are presented for only two scenarios with small ($N_1 = 120$) and large ($N_1 = 250$) population size, with a gradient spatial covariate, $\beta_w = 1$ and $\beta_h = -1$.

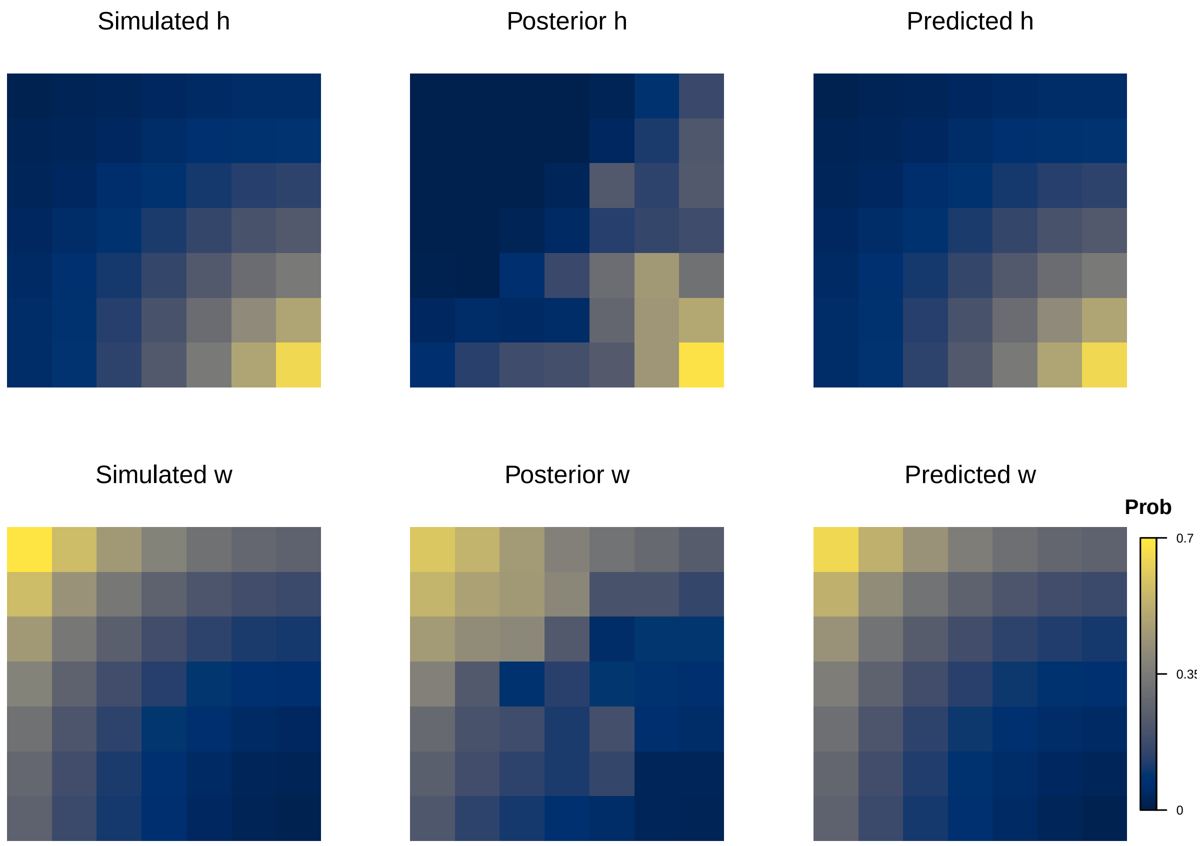

Figure S 2: Example of the spatial pattern in mortality for h (top row) and w (bottom row) used to simulate the data (Simulated), estimated by the model using the average individual posterior s and z (Posterior), and predicted by the model using eqn 3 and 4 (Predicted). Note that w and h are on the probability (Prob) scale. Maps were computed using one replicated dataset and the second survival transition for the 'Posterior' maps.
