# Supplementary material for: Estimating spatially variable and density‐dependent survival using open‐population spatial capture–recapture models
Source: Ecology. 2023 Jan 5;104(2):e3934. doi: 10.1002/ecy.3934 (PMC10078101; doi:10.1002/ecy.3934)
Supplement: Supplementary file 2 — Appendix S2 [file ECY-104-0-s002.pdf]

Appendix S2:
Estimating spatially variable and density-dependent survival using open-population spatial capture–recapture models

Cyril Milleret[1], Soumen Dey[1], Pierre Dupont[1], Henrik Brøseth[2], Daniel Turek[3], Perry de Valpine[4], Richard Bischof[1]

[1] Faculty of Environmental Sciences and Natural Resource Management, Norwegian University of Life Sciences, NO-1432 As, Norway.
[2]Norwegian Institute for Nature Research (NINA), NO-7485 Trondheim, Norway
[3] Department of Mathematics and Statistics, Williams College, Williamstown, MA 01267, USA.
[4] Department of Environmental Science, Policy and Management, University of California Berkeley, Berkeley, USA.

Journal name: Ecology

Detailed description of the wolverine empirical example

1. *Wolverine data*

Wolverines are monitored annually by Swedish and Norwegian authorities using both searches of active natal dens (to obtain a minimum count of the number of born litters), non-invasive genetic sampling (NGS) and dead recoveries (Gervasi et al. 2016). Each year, mostly in late winter and spring, wardens from the State Nature Inspectorate in Norway and from the Environmental Protection Agency in Sweden search for natal dens and collect scat and hair samples along wolverine tracks on snow, covering the entire distribution range of the species in Scandinavia. Search tracks were recorded with GPS by the authorities and constitute our main covariate to quantify spatio-temporal variation in search effort. Non-invasive genetic samples were collected between December and June from individuals (≥1 year old), but not from young of the year born in February-March. For further details on the DNA analysis procedure see (Flagstad et al. 2004, Brøseth et al. 2010, Gervasi et al. 2016) and for details on monitoring of dens see (Gervasi et al. 2014). For the sake of demonstrating the method presented in this paper, we only considered detections collected South of the E14 highway. The

E14 crosses the Scandinavian Peninsula from east to west and constitutes a clear barrier for the wolverine population, as little movement of individuals in and out of that area are observed (Gervasi et al. 2016). In total, we used 2443 genotyped samples of female wolverines collected during seven consecutive winters (2014-2020; Appendix S4 Table S1-2). The Scandinavian wolverine population is transboundary, with two strikingly different harvest regimes across the national border between Sweden and Norway due to differences in conflict potential with free-ranging semi-domestic sheep and legal regulations (Bischof et al. 2020b). Sweden has a very restrictive harvest of wolverines, while Norway has been much more liberal (Gervasi et al. 2019). Wolverines in Norway are exposed to an annual license hunt as well as an extensive damage control program. Thus, we also obtained recovery locations and genetic identification data from 118 females legally culled during the study period. Another eight individuals were found dead due to other causes (e.g., poaching, car collision; Table S3).

2. *OPSCR model*

To estimate survival probabilities of wolverines, we built a Bayesian hierarchical OPSCR model to estimate spatially-explicit causes of mortality as described in paragraph 2.1 of the main text, but with some customization linked to the population and monitoring regime of the wolverine in Scandinavia (Ergon and Gardner 2014, Royle et al. 2014, Bischof et al. 2016, 2020b, Milleret et al. 2020).

The demographic model We used the same demographic model as described in paragraph 2.1.2 of the main text. Individuals considered alive could either die from culling and transition to $z_{i,t}=3$ with probability h_t, or die from other causes and transition to $z_{i,t} = 4$ with probability w_t, so that $z_{i,t} \sim dcat(0, \Phi_t, h_t, w_t)$, where $\Phi_t = 1 - h_t - w_t$. All legal culling mortality events were reported, but most other mortality remains cryptic. Imperfect detection of non-culling mortality

prevents further breakdown of estimates by cause-specific mortality, such as natural, traffic, and poaching deaths.

We created a binary covariate (*Country*) with value 0 for habitat cells in Norway and 1 for habitat cells in Sweden. We modeled individual mortality hazard rates as a function of individual activity center location. We modelled spatial variation in culling β_h and other causes β_w of wolverine mortalities as an additive function of country (Norway = 0, and Sweden=1) and local density at time t ($\beta_{Dens_{\mathrm{w}}}$, $\beta_{Dens_{\mathrm{h}}}$):

$$log(m_{H_{i,t}}) = \log(m_{0h}) + \beta_h * Country_{s_{i,t}} + \beta_{Dens_{\mathrm{h}}} * \log(d_{t_{s_{i,t}}} + 1) \qquad \text{eqn S1}$$

$$log(m_{W_{i,t}}) = \log(m_{0w}) + \beta_w * Country_{s_{i,t}} + \beta_{Dens_{\mathrm{w}}} * \log(d_{t_{s_{i,t}}} + 1) \qquad \text{eqn S2}$$

For the illustration of the method, we did not account for other sources of variation in mortality. However, it could be possible to estimate temporal variation in mortality, for example by using annually varying slope parameters (β_h, β_w) or intercepts (m_{0h}, m_{0w}).

The movement model We used an inhomogeneous point process to model the distribution of individual activity centers (ACs) with a spatial intensity $\lambda(s)$ (where s is a vector of x and y spatial coordinates of ACs) (Zhang et al. 2020). We discretized the habitat into a grid of 20 x 20 km habitat cells to allow the placement of individual AC $s_{i,t}$ as a function of a spatial covariate X (main text, Figure 3). The initial individual AC locations $s_{i,1}$ were conditional on *X*:

$$\lambda(s_{i,1}) = e^{\beta_{AC} X(s_{i,1})}, \qquad \text{eqn S3}$$

where $X(s_{i,1})$ is the value of the spatial covariate at $s_{i,1}$ and β_{AC} the slope parameter describing the relationship between the habitat covariate and density. Here, *X* was defined as the average number of known wolverine dens as a proxy for wolverine density (Bischof et al. 2020b,

Milleret et al. 2021). For t>1, the probability density of $s_{i,t}$, was conditional on the spatial covariate *X* and the Euclidean distance to $s_{i,t-1}$:

$$\lambda(s_{i,t}|s_{i,t-1},\tau) \propto e^{-\frac{\|s_{i,t}-s_{i,t-1}\|^2}{2\tau^2}}.e^{\beta_{AC}X(s_{i,t})}, \quad \text{eqn S4}$$

where τ is the standard deviation of a bivariate normal distribution centered on $s_{i,t-1}$. Under this specification, movement is described as an isotropic Gaussian random walk weighted by the spatial covariate *X* (Ergon and Gardner 2014, Bischof et al. 2020b, Zhang et al. 2020), and τ regulates the distance that individuals are likely to move between years. Such movement feature has been shown to help distinguish between mortality and emigration (Ergon and Gardner 2014, Gardner et al. 2018).

The observation model We used the half-normal function to model detection probability such as described in equation 7 of the main text:

$$p_{i,j,t} = p_{0_{i,j,t}}.exp\ (\frac{-D^2_{i,j,t}}{2\sigma^2}) \quad \text{eqn S5}$$

To account for individual, spatial, and temporal heterogeneity in detection probability, we modelled the individual-, detector-, and year-specific baseline detection probability ($p_{0_{i,j,t}}$.) as a function of the:

1) Length of GPS search tracks logged by wardens within each detector grid cell in each monitoring period (*Tracks*).

2) Average distance from the nearest road (*Roads*): the distance from each detector to the closest road (1:100,000, the Swedish mapping, cadastral and land registration authority; N50 kartdata, the Norwegian Mapping Authority,). This variable represents accessibility, which we predicted to facilitate detectability.

3) Yearly average percentage of snow cover in each detector grid cell (MODIS at 0.1 degrees resolution, www.neo.sci.gsfc.nasa.gov, accessed 2019-10-11) between December 1–June 31 (*Snow*). As wolverine NGS during winter relies heavily on the presence of snow, we predicted that greater snow cover increases detectability.

4) Indicator of whether an individual was detected or not during the previous monitoring season (*PrevDetection*). Previous detection could be expected to positively influence the probability of being detected at subsequent occasions (Gervasi et al. 2014).

5) Country and year. We estimated yearly baseline detection probabilities ($p_{0Intercept}$) for each country to account for differences between the national monitoring regimes.

$$logit\left(p_{0_{i,j,t}}\right) = p_{0Intercept_t\,country[j]} + \beta_{tracks} * Tracks_{j,t} + \beta_{snow} * Snow_{j,t} + \beta_{roads} * Roads_{j,t} + \beta_{prevDetection} * PrevDetection_{i,t} \quad \text{eqn S6}$$

We assigned detections to the closest detectors defined as the center of a 10 x 10 km detector grid cells. The detector grid cells were further subdivided into 25 subdetectors (2 km resolution) (Milleret et al. 2018), and each detection was assigned to the closest subdetector. We then modeled the frequency of subdetectors with ≥ 1 detection $y_{i,j,t}$ as a binomial response with sample size K_j, the number of subdetectors in grid cell *j* that overlapped with the habitat (Milleret et al. 2018):

$$y_{i,j,t} \sim Binomial(p_{i,j,t} * I(z_{i,t} = 2),\ K_j) \quad \textit{eqn S7}$$

where $I(z_{i,t} = 2)$ is an indicator function used to condition detection on the individual being alive. This design allowed us to reduce the number of detectors *j* involved in the calculation of $p_{i,j,t}$ while retaining as many binary detections as possible (Milleret et al. 2018). In addition,

we added a 60 km buffer (>6σ,(Sun et al. 2014)) around the detector grid to allow the placement of AC, and therefore the movement of individuals in and out of the trapping grid (Royle et al. 2014, Bischof et al. 2020b).

Dead recovery model We followed the procedure described in paragraph 2.1.4 in the main text. We modelled dead recoveries within the entire habitat (main text, Figure 3). Only the observation of dead recoveries that were caused by culling were modelled using *eqn* 9 in the main text. This means that we did not use the spatial information contained in the 8 dead recoveries caused by other causes than culling. Dead recovery information from all causes other than culling was only used to set the individual state of individuals $z_{i,t}$ to 4 when the mortality event occurred.

We performed several assessments of SCR/OPSCR model robustness throughout the development process that led to the models used in the analysis. Based on simulations, we assessed robustness to nonindependence between observations (Bischof et al. 2020a), spatial aggregation of detection information (Milleret et al. 2018), population closure (Dupont et al. 2019), ignoring spatial heterogeneity in detection probabilities (Moqanaki et al. 2020) or mis-specifying the detection function (Dey et al. 2021). However, while numerous goodness-of-fit (GOF) tests for non-spatial capture–recapture models exist (Pradel et al. 2005), there is a paucity of robust GOF tests for Bayesian SCR and especially OPSCR (Dey et al. 2021).

Table S1. Annual number of female wolverine non-invasive genetic samples included in the analysis. Numbers are reported by country. We included only samples collected within the study area during the primary monitoring period (Dec 1 - Jun 31) between 2014 (2014/15) and 2020 (2020/21).

	2014	2015	2016	2017	2018	2019	2020	Total
**Norway**	122	180	170	180	272	238	239	1401
**Sweden**	76	90	104	180	175	162	255	1042
**Total**	198	270	274	360	447	400	494	2443

Table S2. Annual number of individual wolverines detected via non-invasive genetic sampling and included in the analysis. Numbers are reported by country. We included only samples collected within the study area during the primary monitoring period (Dec 1 - Jun 31) between 2014 (2014/15) and 2020 (2020/21). Some individuals were detected in both countries during the same year, hence the sum of the national counts can exceed the total number of unique individuals detected.

	2014	2015	2016	2017	2018	2019	2020	Total
**Norway**	49	65	59	62	87	71	72	209
**Sweden**	40	49	56	75	74	82	108	237
**Total**	88	113	114	137	160	150	176	429

Table S3. Number of dead recoveries (culling and other) of wolverines in Scandinavia between 2014 and 2020. Numbers are reported by country. We included all dead recoveries samples collected within the study area between 2014 (2014/15) and 2020 (2020/21).

	2014	2015	2016	2017	2018	2019	2020	Total
**Norway**	22	10	17	10	25	17	11	112
**Sweden**	5	2	1	1	1	3	1	14
**Total**	27	12	18	11	26	20	12	126

Figures

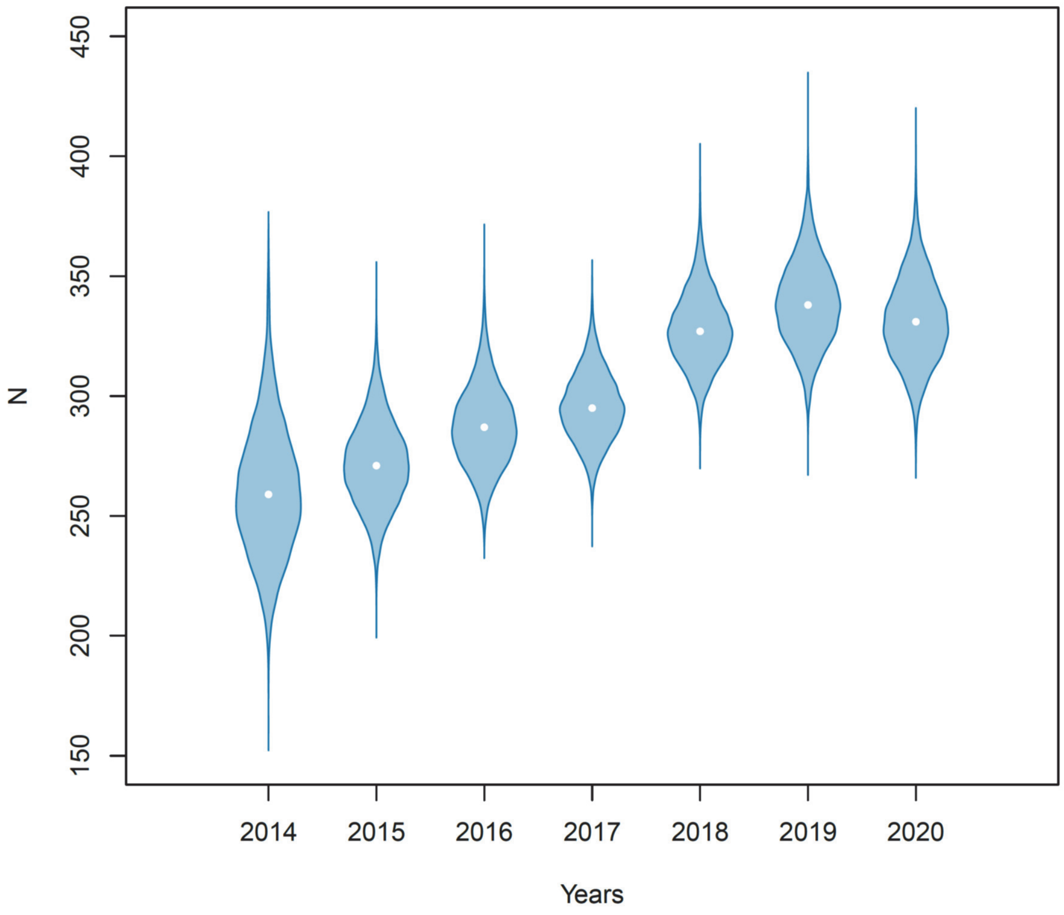

Figure S1. Yearly abundance of female wolverines within the entire habitat from 2014 to 2020 (main text, Figure 3). Violins show the posterior distribution and points the median estimates.

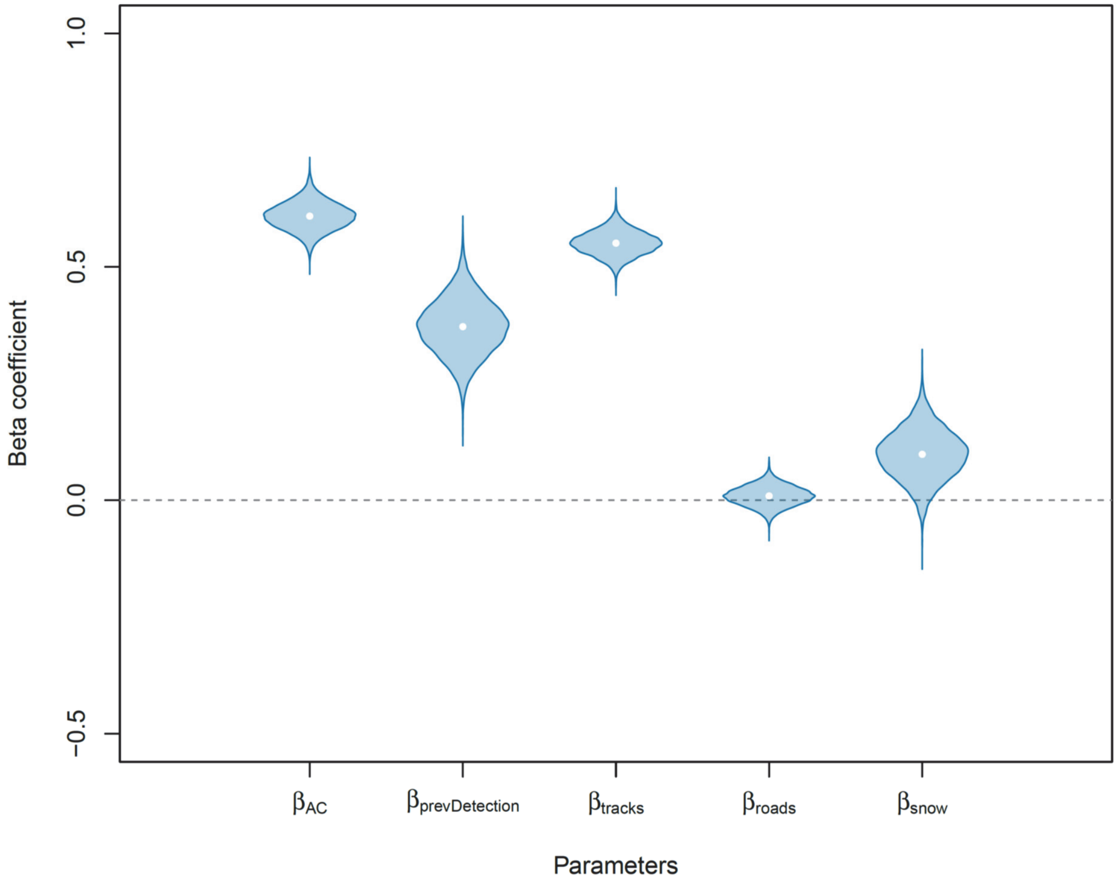

Figure S2. Effect of the average number of known wolverine dens of wolverine density (β_{AC}; section 1.2 of the appendix S4) and effects of the different covariates considered on baseline detection probability (p0; $\beta_{PrevDetection}$, β_{tracks}, β_{roads}, β_{snow}; see appendix S4). Violins show the posterior distribution and points the median estimates.

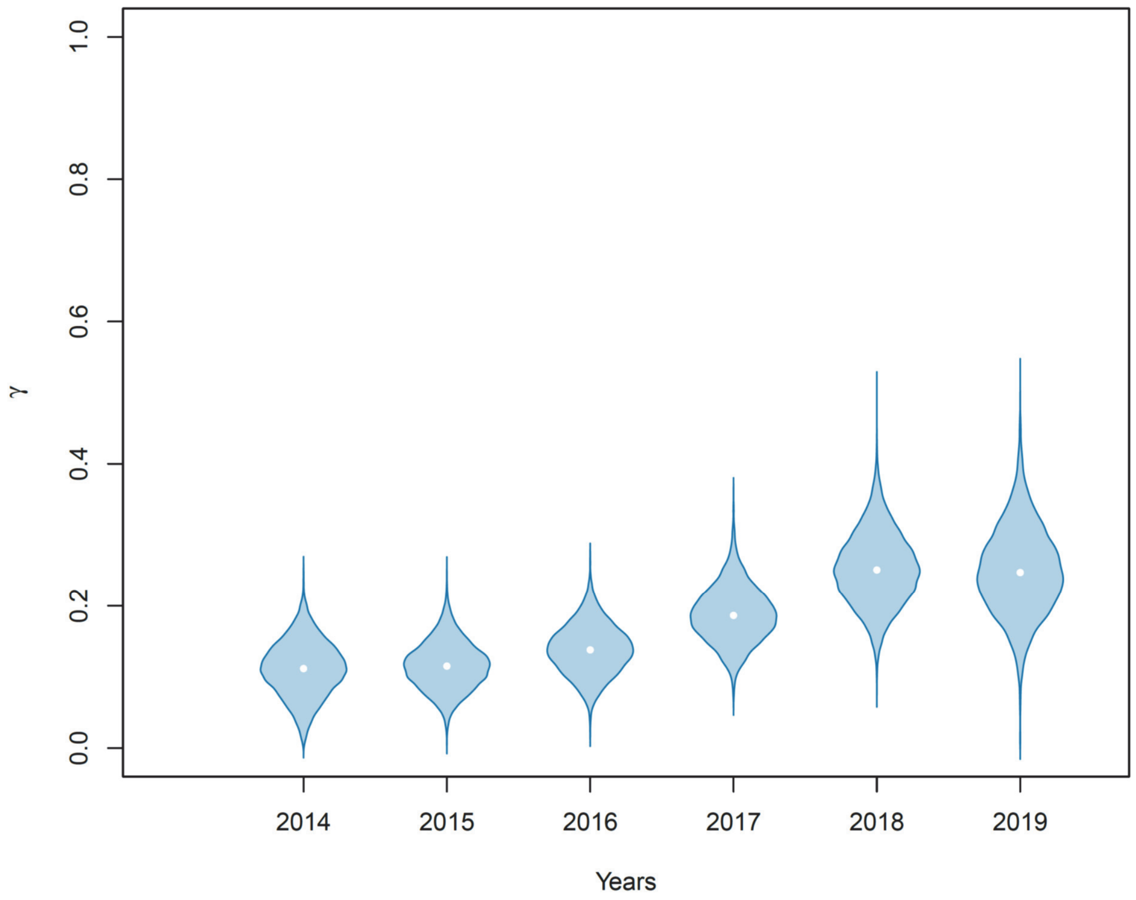

Figure S3. Yearly γ representing the individual transition probability from the state “unborn” to “alive” within the entire habitat from 2014 (2014/15) to 2020 (2020/21).. Violins show the posterior distribution and points the median estimates.

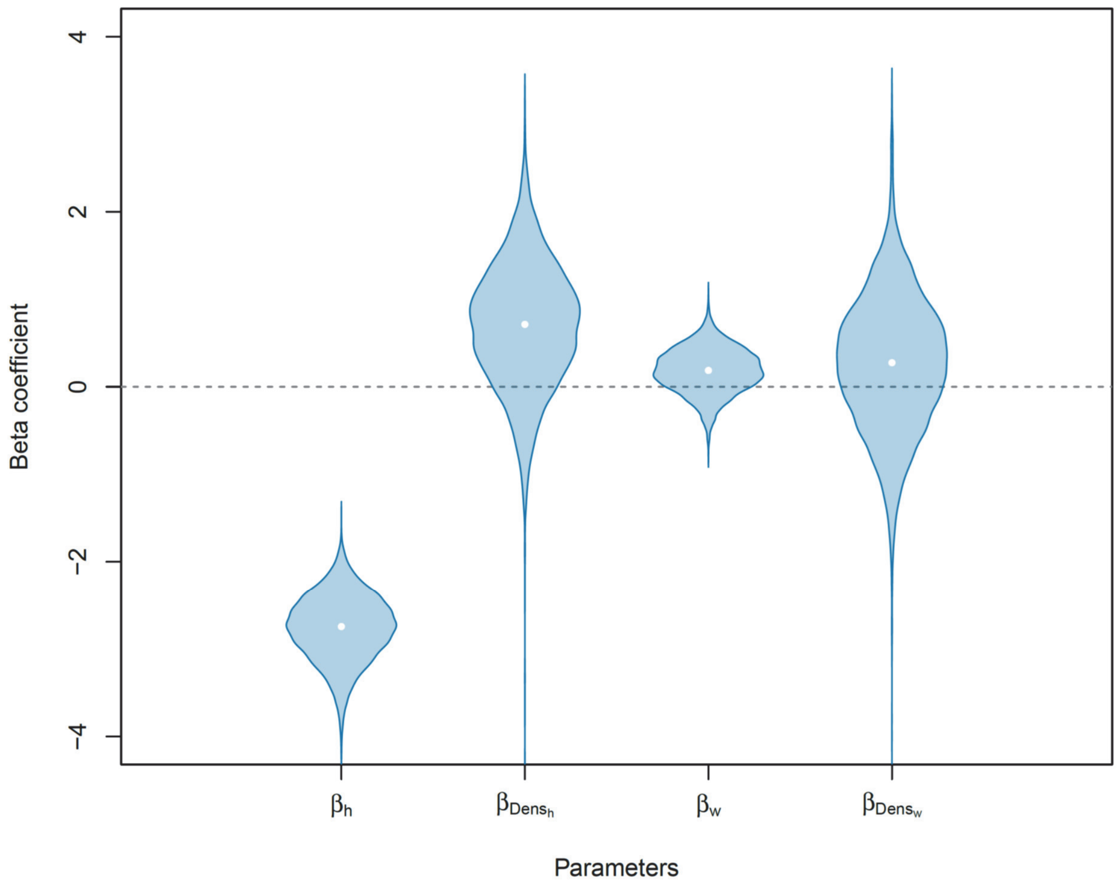

Figure S4. Estimates of the effect of the country (Norway=0, Sweden =1) on hazard mortality rates due culling (β_h) and other causes (β_w). Estimates of the effect of the wolverine density on hazard mortality rates due culling (β_{Dens_h}) and other causes (β_{Dens_w}). A positive beta coefficient denotes an increase in mortality rates with an increase in the covariate value. Violins show the posterior distribution and points the median estimates.

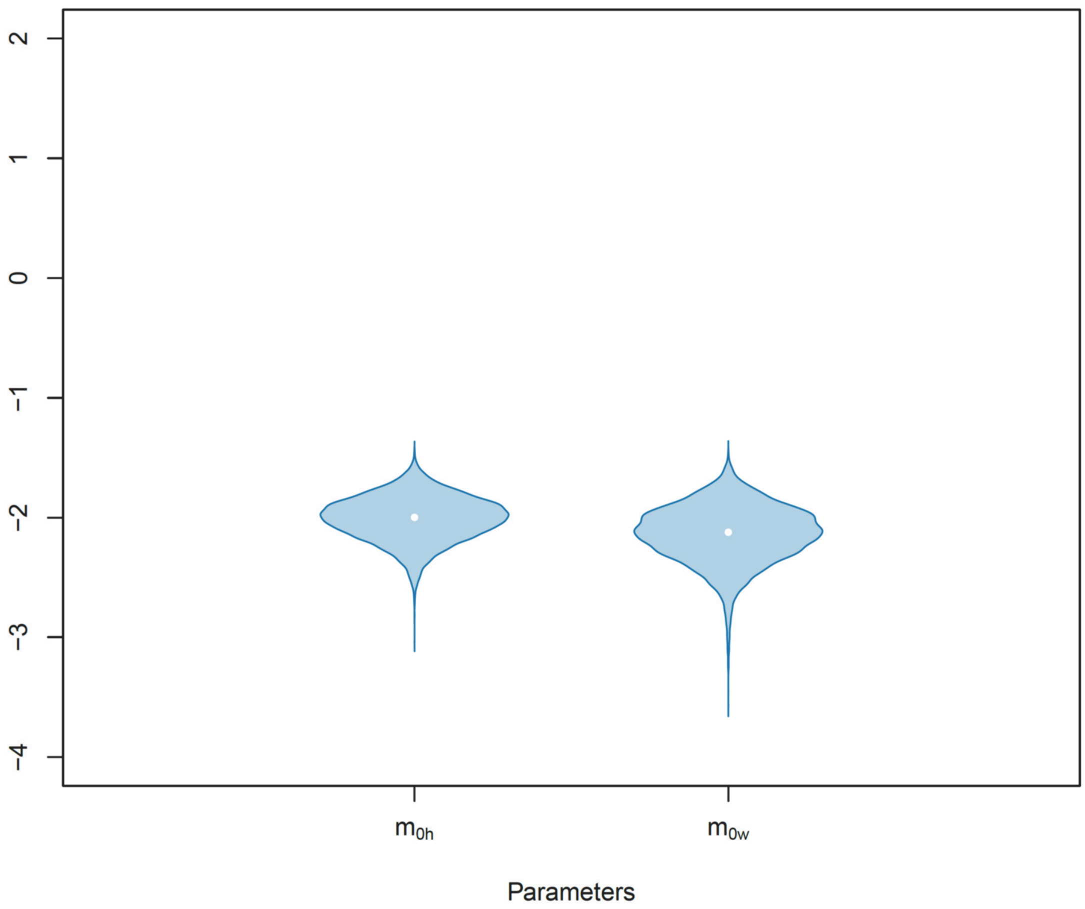

Figure S5. Estimates of the baseline hazard mortality rates of culling (m_{0_h}) and other causes (m_{0_w}) for female wolverines within the study area. Violins show the posterior distribution and points the median estimates.

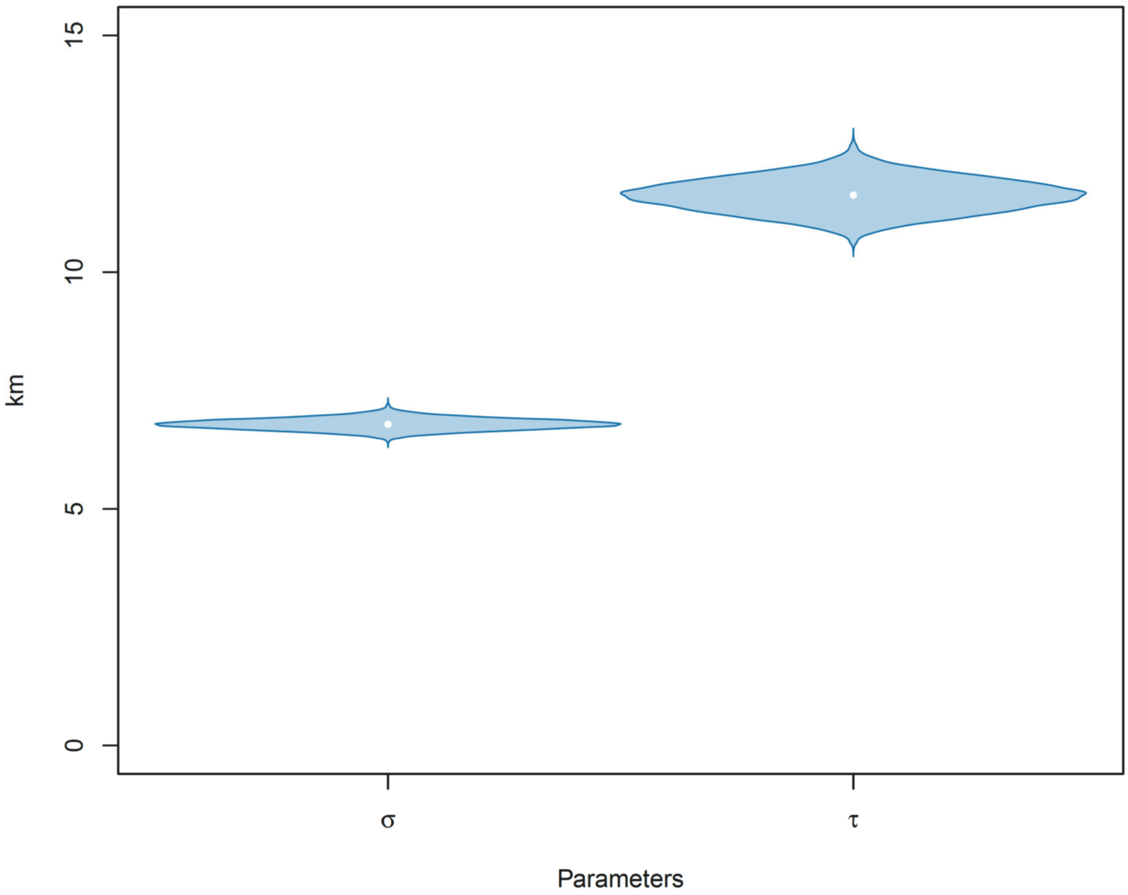

Figure S6. Estimates of the scale parameter σ of the detection function representing movement of individuals around their activity centers (AC) within the sampling periods, and scale parameter τ of the bivariate normal representing movement of AC locations between sampling periods. Violins show the posterior distribution estimates (points: median estimates; solid colors: 95% credible interval). Violins show the posterior distribution and points the median estimates.

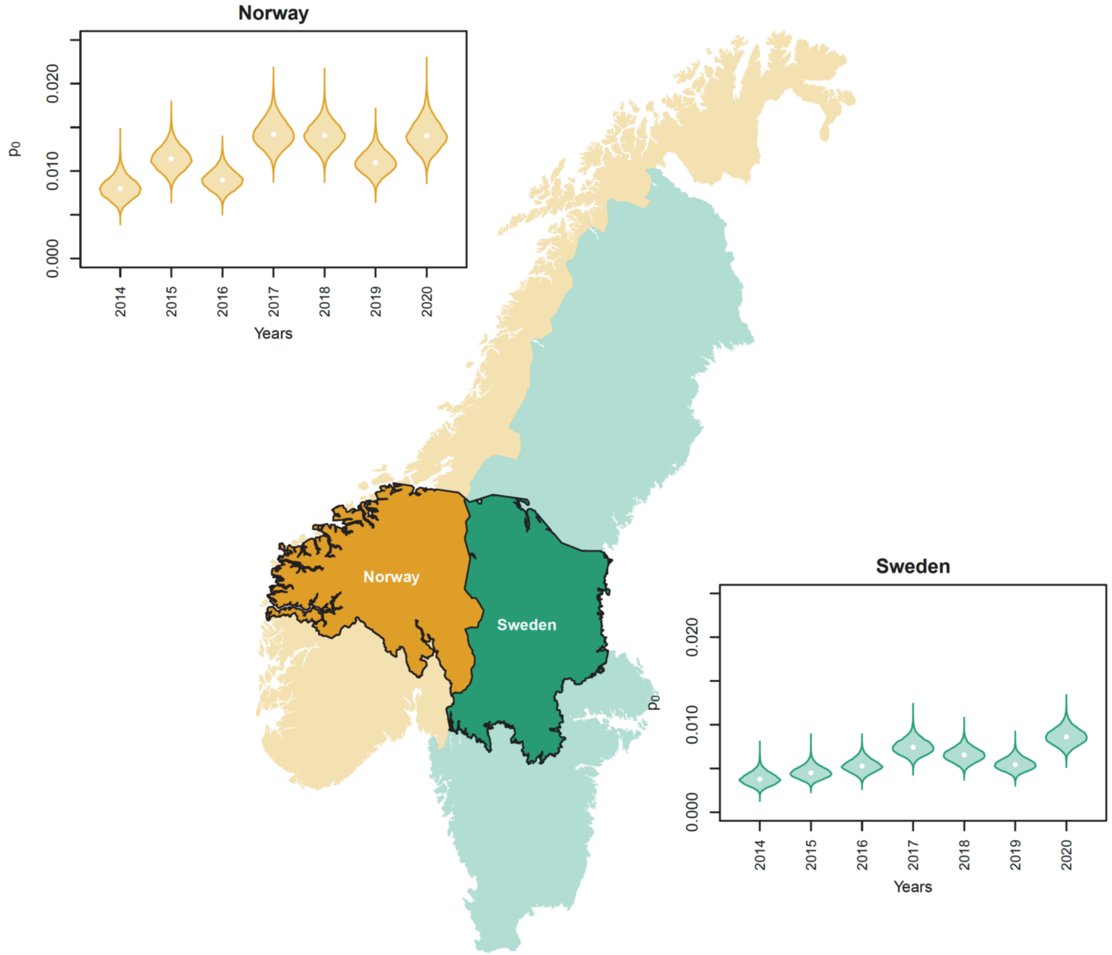

Figure S7. Estimates of the baseline detection probability (p_0) of females wolverines estimated separately for each year and for Norway and Sweden within the study area. Violins show the posterior distribution and points the median estimates.

References

Bischof, R., H. Brøseth, and O. Gimenez. 2016. Wildlife in a Politically Divided World: Insularism Inflates Estimates of Brown Bear Abundance. Conservation Letters 9:122–130.

Bischof, R., P. Dupont, C. Milleret, J. Chipperfield, and J. A. Royle. 2020a. Consequences of ignoring group association in spatial capture-recapture analysis. Wildlife Biology 2020:wlb.00649.

Bischof, R., C. Milleret, P. Dupont, J. Chipperfield, M. Tourani, A. Ordiz, P. de Valpine, D. Turek, J. A. Royle, O. Gimenez, Ø. Flagstad, M. Akesson, L. Svensson, H. Brøseth, and J. Kindberg. 2020b. Estimating and forecasting spatial population dynamics of apex predators using transnational genetic monitoring. Proceedings of the National Academy of Sciences.

Brøseth, H., Ø. Flagstad, C. Wärdig, M. Johansson, and H. Ellegren. 2010. Large-scale noninvasive genetic monitoring of wolverines using scats reveals density dependent adult survival. Biological Conservation 143:113–120.

Dey, S., R. Bischof, P. P. A. Dupont, and C. Milleret. 2021. Does the punishment fit the crime? Consequences and diagnosis of misspecified detection functions in Bayesian spatial capture-recapture modelling. bioRxiv.

Dupont, P., C. Milleret, O. Gimenez, and R. Bischof. 2019. Population closure and the bias-precision trade-off in spatial capture–recapture. Methods in Ecology and Evolution 10:661–672.

Ergon, T., and B. Gardner. 2014. Separating mortality and emigration: modelling space use, dispersal and survival with robust-design spatial capture-recapture data. Methods in Ecology and Evolution 5:1327–1336.

Flagstad, Ø., E. V. A. Hedmark, A. Landa, H. Brøseth, J. Persson, R. Andersen, P. Segerström, and H. Ellegren. 2004. Colonization history and noninvasive monitoring of a reestablished wolverine population. Conservation Biology 18:676–688.

Gardner, B., R. Sollmann, N. S. Kumar, D. Jathanna, and K. U. Karanth. 2018. State space and movement specification in open population spatial capture-recapture models. Ecology and Evolution 8.

Gervasi, V., H. Brøseth, O. Gimenez, E. B. Nilsen, and J. D. C. Linnell. 2014. The risks of learning: confounding detection and demographic trend when using count-based indices for population monitoring. Ecology and Evolution 4:4637–4648.

Gervasi, V., H. Brøseth, O. Gimenez, E. B. Nilsen, J. Odden, Ø. Flagstad, and J. D. C. Linnell. 2016. Sharing data improves monitoring of trans-boundary populations: the case of wolverines in central Scandinavia. Wildlife Biology 22:95–106.

Gervasi, V., J. D. C. Linnell, H. Brøseth, and O. Gimenez. 2019. Failure to coordinate management in transboundary populations hinders the achievement of national management goals: The case of wolverines in Scandinavia. Journal of Applied Ecology 0.

Milleret, C., R. Bischof, P. Dupont, H. Brøseth, J. Odden, and J. Mattisson. 2021. GPS collars have an apparent positive effect on the survival of a large carnivore. Biology Letters 17:20210128.

Milleret, C., P. Dupont, H. Brøseth, J. Kindberg, A. Royle J., and R. Bischof. 2018. Using partial aggregation in spatial capture recapture. Methods in Ecology and Evolution 0.

Milleret, C., P. Dupont, J. Chipperfield, D. Turek, H. Brøseth, O. Gimenez, P. de Valpine, and R.

Bischof. 2020. Estimating abundance with interruptions in data collection using open population spatial capture-recapture models. Ecosphere 11:e03172.

Moqanaki, E. M., C. Milleret, M. Tourani, P. Dupont, and R. Bischof. 2020. Consequences of ignoring variable and spatially-autocorrelated detection probability in spatial capture-recapture. bioRxiv.

Pradel, R., O. Gimenez, and J.-D. Lebreton. 2005. Principles and interest of GOF tests for multistate capture--recapture models. Animal Biodiversity and Conservation 28:189–204.

Royle, J. A., R. B. Chandler, R. Sollmann, and B. Gardner. 2014. Spatial Capture-Recapture. Academic Press.

Sun, C. C., A. K. Fuller, and J. A. Royle. 2014. Trap Configuration and Spacing Influences Parameter Estimates in Spatial Capture-Recapture Models. PLOS ONE 9:1–9.

Zhang, W., J. D. Chipperfield, J. B. Illian, P. Dupont, C. Milleret, P. de Valpine, and R. Bischof. 2020. A hierarchical point process model for spatial capture-recapture data. bioRxiv.
